# Multifunctional Metal Halide Perovskite‐Modified Aqueous Electrolytes for Zinc Metal Batteries

**DOI:** 10.1002/advs.202509417

**Published:** 2025-07-28

**Authors:** Tong Yang, Jiahui Lu, Minh Tam Hoang, Yang Yang, Shijian Wang, Yong Chen, Yongyue Yu, Yan Wang, Timing Fang, Bing Sun, Guoxiu Wang, Hongxia Wang

**Affiliations:** ^1^ School of Chemistry and Physics Queensland University of Technology 2 George St Brisbane City QLD 4000 Australia; ^2^ School of Mathematical and Physical Science University of Technology Sydney 15 Broadway Ultimo NSW 2007 Australia; ^3^ College of Chemistry and Chemical Engineering Qingdao University Qingdao Shandong 266071 P. R. China

**Keywords:** aqueous zinc metal batteries, electrode interface, electrolyte additives, electrolyte solvation, metal halide perovskite

## Abstract

The performance of Zn metal batteries (ZMBs) is significantly hindered by the poor cycling stability and dendrite growth of Zn metal anodes. Herein, Cs_2_SnCl_6_ is introduced, a lead‐free metal halide double perovskite, as a multifunctional electrolyte additive to address the challenges of Zn anodes. Utilizing a combination of molecular dynamics simulations, COMSOL simulations, and various characterization techniques, it is demonstrated that Cl^−^, Sn^4+^, and Cs^+^ ions generated from partial hydrolysis of Cs_2_SnCl_6_ in the 2 m ZnSO_4_ electrolyte can optimize the electrolyte solvation structures, suppress side reactions, facilitate Zn nucleation process, and modulate Zn deposition behavior. As a result, Zn||Zn symmetric cells with Cs_2_SnCl_6_‐enhanced electrolyte achieve remarkable cycling stability over 5000 h at 1 mA cm^−2^, while the full cell also shows a capacity retention of 99.96% after 1000 cycles. This work provides insights into electrolyte‐driven interface modulation strategies for next‐generation aqueous ZMBs.

## Graphical abstract

Multifunctional metal halide perovskite‐modified aqueous electrolytes for zinc metal batteries introducing Cs_2_SnCl_6_ as an electrolyte additive significantly enhances battery performances by regulating solvent structure, stabilizing pH, and restricting dendrite growth. The Cs_2_SnCl_6_ modified electrolyte promotes uniform zinc plating, achieves exceptional cycling stability, and improves Zn||V_2_O_5_ full‐cell performance, demonstrating a promising approach to achieve practical zinc metal batteries.

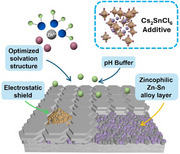

## 1 Introduction

Lithium‐ion (Li‐ion) batteries dominate the current energy storage market due to their high energy density and efficiency. However, they still face challenges such as high costs, toxicity, and safety risks.^[^ ^1^ ^]^ In contrast, aqueous Zn metal batteries (ZMBs) have emerged as a promising alternative, acclaimed for their environmental sustainability, improved safety, and abundant resources.^[^ ^2^, ^3^, ^4^, ^5^ ^]^ Nevertheless, ZMBs also face their own challenges. They are prone to dendritic formations due to inhomogeneous Zn^2+^ ion distributions at the interface between the Zn anode and the electrolyte. Additionally, self‐corrosion and unwanted hydrogen evolution reactions (HER) compromise their inherent thermodynamic stability. Another critical issue is the accumulation of weakly adherent passivation layers (e.g., Zn_4_SO_4_(OH)_6_.5H_2_O) on Zn anodes during battery cycling, leading to increased interfacial resistance and the loss of active electrode materials. These challenges contribute to reduced cycling reversibility, accelerated capacity degradation, and ultimately, premature battery failure.^[^ ^6^, ^7^ ^]^

Various strategies have been proposed to address the challenges of ZMBs, including advanced electrolyte formulations,^[^ ^5^, ^8^ ^]^ interfacial engineering,^[^ ^9^, ^10^, ^11^ ^]^ the development of 3D and porous electrode structures, and the use of ion‐selective membranes and separators.^[^ ^12^ ^]^ Among these strategies, electrolyte engineering stands out as an effective, cost‐efficient, and straightforward method to improve the stability of the electrolyte‐anode interface.^[^ ^13^ ^]^ For instance, Huang et al. enhanced the compatibility of Zn metal anodes and stabilized the pH value of electrolytes by incorporating poly‐L‐glutamic acid as a polymer additive.^[^ ^14^ ^]^ Xu et al. reported significant improvements in battery cycling stability by introducing silk fibroin (SF) into the electrolyte, which modifies the solvent structure and forms a self‐healing protective film on the Zn anode.^[^ ^15^ ^]^ Additionally, Wu et al. enhanced the interface electric field uniformity in Zn‐ion batteries by incorporating silicon nanoparticles as electrolyte additives, effectively inhibiting Zn dendritic growth and extending the cycle life.^[^ ^16^ ^]^ To date, a wide range of additives, including proteins, polymers, inorganic substances, and salts, have been explored.^[^ ^17^ ^]^ These additives primarily enhance battery performance by facilitating nucleation processes, optimizing solvation structures, reducing side reactions, stabilizing pH values of the electrolytes, mitigating uneven electric field distribution, and suppressing Zn dendritic growth. However, most additives offer limited functionality, enhancing battery performance from only one or two aspects. Therefore, the discovery of multifunctional additives capable of simultaneously enhancing both the stability and efficiency of Zn metal anodes is crucial for the practical application of ZMBs.

Metal halide perovskites represent a distinguished class of materials owing to their tunable chemical compositions and unique lattice structures. These materials exhibit exceptional light absorption and charge‐carrier mobility, typically configured in an ABX_3_ lattice configuration, where “A” is a monovalent cation, “B” a divalent metal cation, and “X” a halide.^[^ ^18^, ^19^ ^]^ Their compact lattice structure and remarkable compositional flexibility make metal halide perovskites highly promising for various applications, including Zn‐based batteries. For instance, Wang et al. introduced a low‐dimensional hybrid perovskite, 4,4′‐trimethylenedipyridinium lead iodide/bromide (TmdpPb_2_), as a cathode material for Zn‐ion batteries.^[^ ^20^ ^]^ Leveraging the intrinsic halide exchange capabilities of the perovskite structure, these cathode materials demonstrated remarkable longevity and efficiency, sustaining 400 cycles at 3.2 A g^−1^ with an average Coulombic efficiency of 99%. Historically, research on metal halide perovskites in Zn‐ion batteries has primarily focused on their use as standalone cathode materials or as components of composite cathodes. However, this conventional application leaves significant research potential unexplored. In particular, the role of metal halide perovskite compounds as electrolyte additives for enhancing Zn‐based battery performance remains an untapped research area.

Herein, we introduce a lead‐free double‐side metal halide perovskite, Cs_2_SnCl_6_, as an environmentally friendly and multifunctional electrolyte additive for high‐performance ZMBs. The partial degradation of this perovskite in the aqueous electrolyte releases various cations and anions. As the key degradation products, halide anions (Cl^−^) play a crucial role in regulating the solvent structure and mitigating side reactions. Meanwhile, the released Sn^4+^ cations facilitate the nucleation process by forming a Zn─Sn alloy in the initial nucleation stage. Additionally, the Cs^+^ cations promote the formation of a positively charged electrostatic shield around the initial growth sites of protuberances, effectively preventing further dendrite growth. Moreover, the degradation process helps to stabilize the pH value of the electrolyte, acting as a buffer during cycling. Integrating the electrolyte with Cs_2_SnCl_6_ into ZMBs significantly enhanced the rate performance and cycle life of both symmetric and full cells, demonstrating its potential as a practical and effective additive for ZMBs.

## 2 Results and Discussion

### 2.1 Electrolyte Characterization

**Scheme 1:**
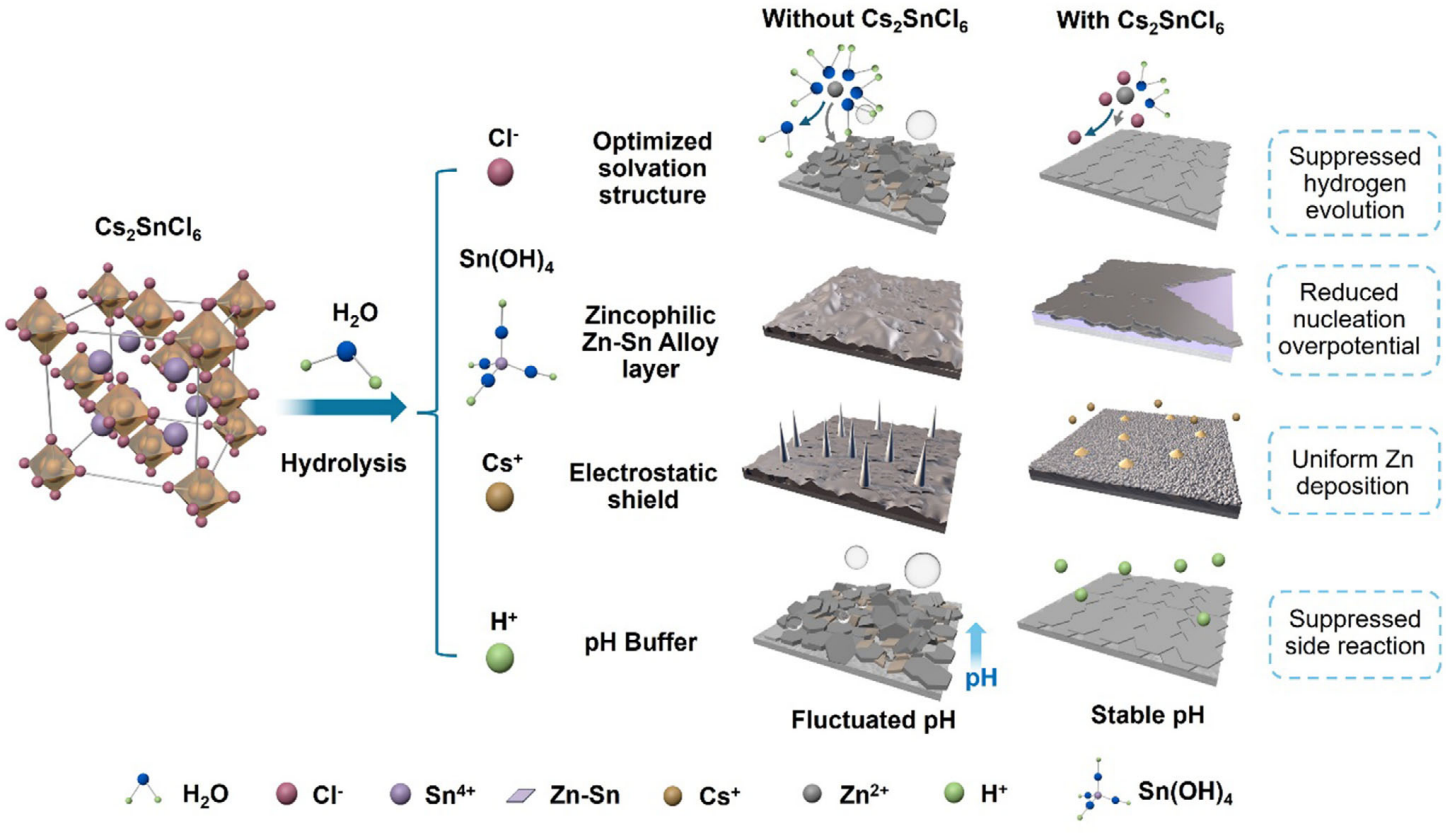
Schematic illustration of the main functions of Cs_2_SnCl_6_ additive in improving the electrochemical performance of Zn metal anodes.

The gradual hydrolysis process of Cs_2_SnCl_6_ in aqueous ZnSO_4_ electrolyte is illustrated in **Scheme** **1**.^[^ ^21^ ^]^ Cs_2_SnCl_6_ features a vacancy‐ordered double perovskite structure, characterized by discrete [SnCl_6_] octahedra. Its enhanced environmental stability can be attributed to the chloride's higher ionic potential, which results from its relatively smaller ionic radius and stronger atomic bonding compared to iodide and bromide.^[^ ^22^ ^]^ The X‐ray diffraction (XRD) pattern of the synthesized Cs_2_SnCl_6_ is presented in Figure S1 (Supporting Information), confirming its cubic structure, which corresponds to the Fm‐3m (225) space group and aligns with the reference pattern (PDF 00‐007‐0197). Scanning electron microscopy (SEM) images reveal that Cs_2_SnCl_6_ particles exhibit an octahedral shape with an average diameter of ≈300 nm (Figure S2, Supporting Information). Upon introducing 0.5 wt.% Cs_2_SnCl_6_ into the 2 m ZnSO_4_ aqueous electrolyte (i.e., 0.5 Cs_2_SnCl_6_ electrolyte), the additive disperses uniformly, as evidenced by the Tyndall effect test (Figure S3, Supporting Information). Cs_2_SnCl_6_ undergoes partial hydrolysis in the 2 m ZnSO_4_ electrolyte, and this process can be described by the following chemical reactions^[^ ^23^ ^]^:

(1) $$Cs_{2}\mathrm{SnC}l_{6}\left(s\right)\to 2Cs^{+}\left(\mathrm{aq}\right)+Sn^{4+}\left(\mathrm{aq}\right)+6Cl^{-}\left(\mathrm{aq}\right)$$

(2) $$Sn^{4+}\left(\mathrm{aq}\right)+4H_{2}O\left(\mathrm{aq}\right)\to \mathrm{Sn}{\left(\mathrm{OH}\right)}_{4}\left(s\right)+4H^{+}\left(\mathrm{aq}\right)$$

Following centrifugation of the electrolyte containing the perovskite additive, an XRD analysis was performed on the obtained precipitate. The XRD results identified the presence of both Cs_2_SnCl_6_ and an amorphous phase, as shown in Figure S4 (Supporting Information). Further investigation revealed that the amorphous signal corresponded to Sn(OH)_4_, as confirmed by matching the XRD pattern to that of the precipitation formed from SnCl_4_.5H_2_O in a 2 m ZnSO_4_ electrolyte (Figure S5, Supporting Information).^[^ ^24^ ^]^ Consequently, the partial hydrolysis of the Cs_2_SnCl_6_ in the electrolyte results in an acidic solution contains unhydrolyzed Cs_2_SnCl_6_ particles, Sn(OH)_4_ precipitates, as well as Cs^+^ and Cl^−^ ions. Each of those components plays a crucial role in enhancing the performance of Zn metal anodes.

**Figure 1:**
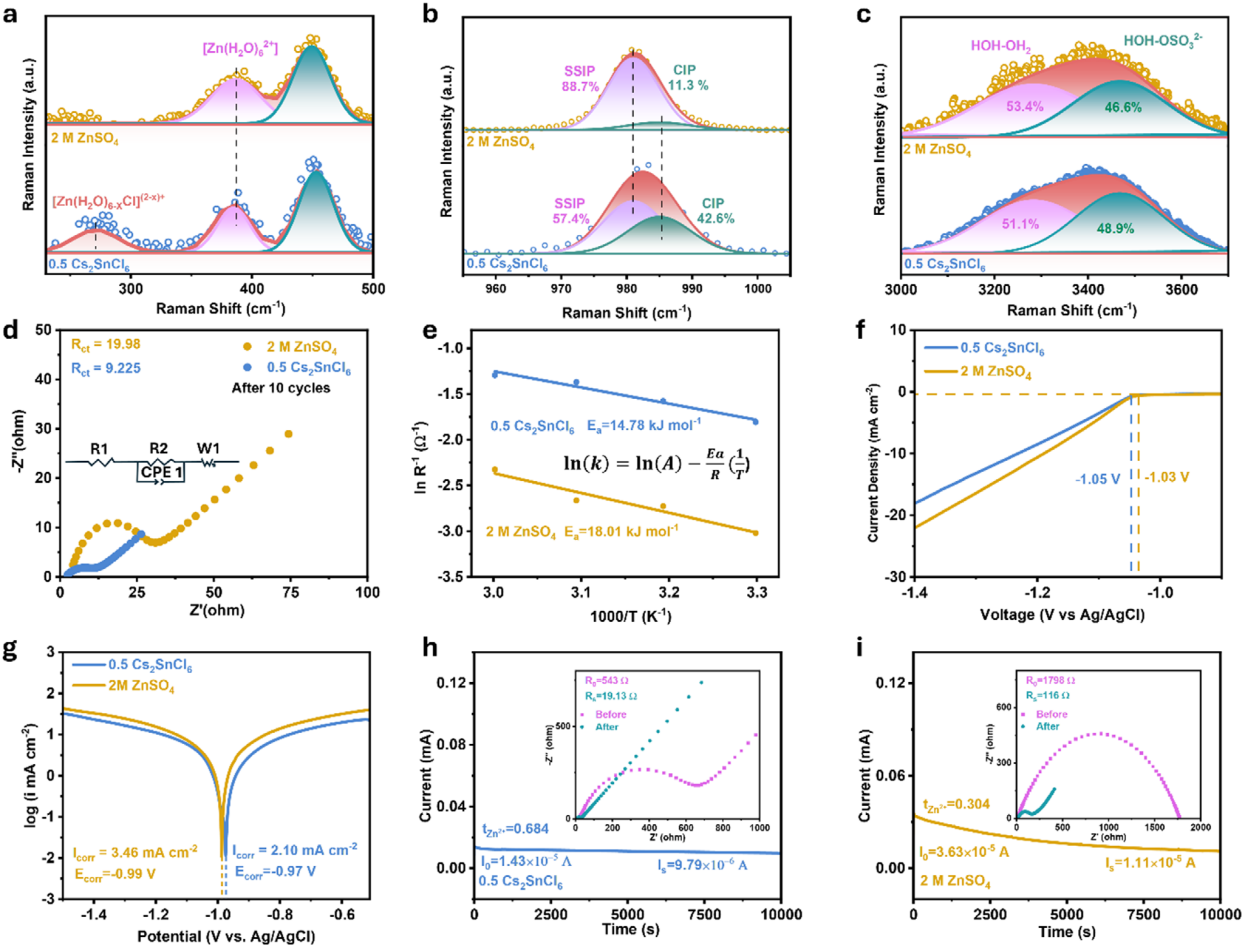
a–c) Raman spectra of the ZnSO_4_ electrolyte and 0.5 Cs_2_SnCl_6_ electrolyte. d) EIS spectra of the Zn||Zn symmetric cells in different electrolytes under 30 °C after 10 cycles Insert: Equivalent Circuit Model. e) Arrhenius plot of different Zn||Zn symmetric cells in different electrolytes. f) LSV plots of different electrolytes at a scan rate of 1.0 mV s^−1^ in the three‐electrode system. g) LSV plots of Zn electrodes in different electrolytes at a scan rate of 2.0 mV s^−1^ in a three‐electrode system. h,i) CA of Zn anodes tested in h) 2 m ZnSO_4_, and i) 0.5 Cs_2_SnCl_6_ electrolytes at a fixed overpotential of 20.0 mV. Inserts in h,i) are the EIS spectra of the cells before and after the CA test.

Raman spectroscopy was used to reveal the solvation structures of the electrolytes with and without Cs_2_SnCl_6_. As shown in **Figure** **1**a, the pristine electrolyte shows a peak at 390 cm^−1^, corresponding to Zn‐OH_2_ vibrations.^[^ ^25^ ^]^ Upon introducing the perovskite, this peak weakened, indicating alterations in the solvation environment of Zn^2+^ ions. Notably, a new broad peak appears at 290 cm^−1^ in the 0.5 Cs_2_SnCl_6_ electrolyte, attributed to the formation of Zn(H_2_O)_6‐x_Cl_x_]^(2−x)+^ complexes, which results from Cl^−^ and Zn^2+^ interactions.^[^ ^26^ ^]^ This shift suggests that Cl^−^ disrupts Zn^2+^‐H_2_O interaction by integrating into the Zn^2+^ solvation shells, thereby mitigating HER during cycling.^[^ ^27^ ^]^ According to the classic Eigen‐Tamm mechanism, when ZnSO_4_ dissolves in water, a fraction of SO_4_^2−^ binds directly to Zn^2+^ through coordination, forming a contact ion pair [Zn^2+^(H_2_O)_5_OSO_3_^2−^] (CIP). Meanwhile, the majority of Zn^2+^ species interact with six water molecules before associating with SO_4_^2−^, resulting in a solvent‐separated ion pair [Zn^2+^(H_2_O)_6_ SO_4_^2−^] (SSIP).^[^ ^28^ ^]^ In the pristine 2 M ZnSO_4_ electrolyte, the distribution of these ion pair species is 88.7% for SSIP and 11.3% for CIP, whereas in the 0.5 Cs_2_SnCl_6_ electrolyte, these values change to 57.4% for SSIP and 42.6% for CIP (Figure 1b). This shift indicates a weakened interaction between Zn^2+^ and SO_4_^2−^ in the modified electrolyte, which is beneficial in preventing SO_4_^2−^ from participating in undesirable side reactions on the Zn anode surface.^[^ ^29^ ^]^ Furthermore, the Raman peaks associated with O─H vibrations shift to higher frequencies in the 0.5 Cs_2_SnCl_6_ electrolyte (Figure 1c). This shift indicates a decrease in the proportion of free water molecules, underscoring the significant impact of perovskite additives on the electrolyte's molecular interactions.^[^ ^30^ ^]^

Molecular dynamics (MD) simulations were performed to evaluate the impact of Cs_2_SnCl_6_ on the Zn^2+^ solvation structure in a 2 m ZnSO_4_ electrolyte. Simulation models were constructed for both cases, with and without Cs_2_SnCl_6_ (Figure S6, Supporting Information). The introduction of Cs_2_SnCl_6_ leads to the release of Cl^−^ ions, which modifies the original [Zn(H_2_O)_6_]^2+^ configuration by integrating into the primary solvation shells of Zn^2+^ ions and partially replacing the coordinated water molecules. As shown in Figure S7 (Supporting Information), the radial distribution function (RDF) reveals Zn─Ow bonds forming at ≈2 Å. Simultaneously, the average coordination number (ACN) between Zn^2+^ ions and water molecules decreases from 5.798 in the pristine ZnSO_4_ electrolyte to 5.452 upon the addition of Cs_2_SnCl_6_, indicating a reduction in water molecules surrounding Zn^2+^. Furthermore, the Zn‐H_2_O interaction energy, originally ≈−630 eV, shifts to −610 eV with the addition of Cs_2_SnCl_6_, demonstrating that the increased interaction energy facilitates the displacement of water by Cl^−^ in the Zn^2+^ solvation shell (Figure S8, Supporting Information).

To further confirm that the solvent structure was modified by the addition of Cs_2_SnCl_6_, we investigated the molecular interactions within the 0.5 Cs_2_SnCl_6_ electrolyte using nuclear magnetic resonance (NMR) (Figure S9, Supporting Information). The ¹H NMR peak for the pristine 2 m ZnSO_4_ electrolyte appears at 4.347 ppm. With the incorporation of Cs_2_SnCl_6_, this peak shifts to a lower position—4.302 ppm for the 0.5 Cs_2_SnCl_6_ electrolyte and 4.279 ppm for the 2 Cs_2_SnCl_6_ electrolyte (2 wt.% Cs_2_SnCl_6_ into the 2 m ZnSO_4_ aqueous electrolyte), indicating an increase in electronic density around the protons of water molecules. The NMR results demonstrate a weakened coordination strength between H_2_O and Zn^2+^, which contributes to suppressing the HER during Zn deposition.^[^ ^31^ ^]^

To evaluate the impact of perovskite additives on the desolvation process, we calculate the activation energies (E_a_) for Zn^2+^ ion deposition using the Arrhenius equation. This analysis involved fitting the impedance spectra, specifically the charge transfer resistance (R_ct_), of Zn||Zn symmetrical batteries under varying temperature conditions. Figure 1d demonstrates the electrochemical impedance spectroscopy (EIS) of symmetrical cells with different electrolytes under 30 °C.^[^ ^32^ ^]^ The calculation details are provided in the supporting information. As shown in Figure 1e, the E_a_ value in the pristine 2 m ZnSO_4_ electrolyte is 18.01 kJ mol^−1^. In contrast, the 0.5 Cs_2_SnCl_6_ electrolyte demonstrates a significantly reduced activation energy of 14.78 kJ mol^−1^. This reduction indicates enhanced kinetics of Zn^2+^ ion transfer at the electrolyte‐electrode interface and a more favorable desolvation process, underscoring the effectiveness of the Cs_2_SnCl_6_ additive.

The anti‐corrosion effect of the Cs_2_SnCl_6_ in the electrolyte was investigated using electrochemical linear sweep voltammetry (LSV). The 0.5 Cs_2_SnCl_6_ electrolyte showed a significant enhancement in mitigating the self‐corrosion reaction at the Zn anode, evidenced by a higher hydrogen evolution potential (−1.05 V versus Ag/AgCl, Figure 1f) and a lower corrosion current density (2.10 mA cm^−2^, Figure 1g) compared to the 2 M ZnSO_4_ electrolyte, which exhibited a hydrogen evolution potential of −1.03 V versus Ag/AgCl and a corrosion current density of 3.46 mA cm^−2^. Additionally, the ionic conductivities are similar among different electrolytes (Figure S10, Supporting Information).

Chronoamperometry (CA) tests were utilized to study the Zn deposition behaviors in Zn||Zn symmetric cells. The Zn^2+^ ion transference number was calculated from the EIS data, using the formula provided in the Supporting Information, based on the steady‐state current and resistance before and after the CA test. The results, shown in Figure 1h,i, demonstrated that the cell with 0.5 Cs_2_SnCl_6_ electrolyte, under a fixed overpotential of 20 mV for 10 000 s, exhibited a high Zn^2+^ ion transfer number of 0.684, surpassing the 0.304 observed in the 2 m ZnSO_4_ electrolyte.^[^ ^33^ ^]^ This increase in ionic transfer number is attributed to the Cl^−^ ions from Cs_2_SnCl_6_, which compete with SO_4_^2−^ for interaction with Zn^2+^, reducing ion pairing and enhancing the number of free Zn^2+^ ions available for conduction.

Zn immersion tests were conducted to evaluate the effects of different electrolytes on reducing self‐corrosion behaviors. The SEM images in Figure S11a,b (seconds) revealed that the Zn anode immersed in 0.5 Cs_2_SnCl_6_ electrolyte exhibited a much smoother surface, whereas the Zn anode in 2 m ZnSO_4_ was covered with loose and uneven deposits. Furthermore, the XRD pattern of the anode from the 2 m ZnSO_4_ electrolyte (Figure S11c, Supporting Information) displayed a stronger signal of the self‐corrosion product, Zn_4_SO_4_(OH)_6_.5H_2_O, further confirming the enhanced stability of the Zn anode in the 0.5 Cs_2_SnCl_6_ electrolyte.

### 2.2 Zn Nucleation Behavior Investigation

**Figure 2:**
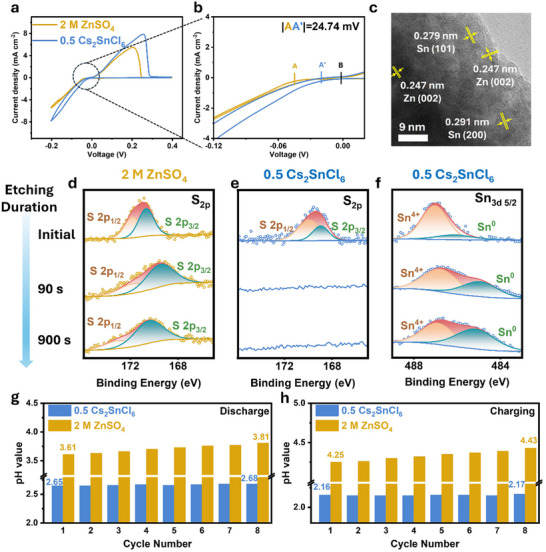
a,b) CV curves of Zn||Cu half‐cell in 2 m ZnSO_4_ and 0.5 Cs_2_SnCl_6_ electrolyte at a scan rate of 1 mV s^−1^ from −0.2 V to 0.4 V. c) TEM images of Zn electrode after 5 cycles under 1 mA cm^−2^, 1 mAh cm^−2^. d–f) XPS spectra of Cu electrode after 5 cycles under 1 mA cm^−2^, 1 mAh cm^−2^ with 2 m ZnSO_4_ and 0.5 Cs_2_SnCl_6_ electrolyte before etching and after etching. g,h) pH value of ZnSO_4_ and 0.5 Cs_2_SnCl_6_ electrolyte of symmetric cell cycled under 5 mA cm^−2^, 1 mAh cm^−2^ at end of each g) discharge processes and h) charge processes.

The nucleation overpotentials (NOP) of Zn deposition in different electrolytes were analyzed using cyclic voltammetry (CV) tests. As shown in **Figure** **2**a,b, the Zn||Zn symmetric cell containing 0.5 Cs_2_SnCl_6_ electrolyte exhibited a lower NOP compared to the cell containing 2 m ZnSO_4_ electrolyte. The reduced NOP indicates a lower energy barrier for Zn nucleation at the anode interface in the 0.5 Cs_2_SnCl_6_ electrolyte, thereby promoting a more uniform Zn deposition.

Given the reduction potentials of 0.15 V (vs SHE, standard hydrogen electrode) for the conversion from Sn^4+^(aq) to Sn^2+^(aq), −0.14 V (vs SHE) for Sn^2+^(aq) to Sn(s), and −0.76 V (versus SHE) for Zn^2+^(aq) to Zn(s),^[^ ^34^, ^35^ ^]^ it is proposed that Sn^4+^ ions in the electrolyte actively participate in the nucleation process during the Zn deposition process. The involvement of Sn element in the nucleation process was confirmed by high‐resolution transmission electron microscopy (HRTEM). As shown in Figure 2c, the surface of the Zn electrode after five cycles at 1 mA cm^−2^ and 1 mAh cm^−2^ displayed a distinct lattice spacing of 0.247 nm, corresponding to the (002) plane of metallic Zn.^[^ ^36^ ^]^ Additionally, the metallic Sn phase was also detected, characterized by lattice spacings of 0.291 nm and 0.279 nm, which matched the Sn (200) and Sn (101) planes, respectively.^[^ ^36^ ^]^ The selected area electron diffraction pattern further confirmed the presence of a Zn─Sn alloy (Figure S12, Supporting Information). The evidence from HRTEM images demonstrates the formation of a Zn─Sn alloy during Zn nucleation process. In addition, zeta potential measurements (Figure S13, Supporting Information) revealed that the stern potential of Zn deposits shifted progressively in the positive direction with the addition of Cs_2_SnCl_6_. This suggests that the 0.5 Cs_2_SnCl_6_ electrolyte reduces the net charge on the surface of Zn metal, facilitating smoother Zn deposition.^[^ ^37^ ^]^

The surface chemistry of Zn deposition layer formed in different electrolytes after cycling was analyzed by X‐ray photoelectron spectroscopy (XPS). The Cu electrodes of the Zn||Cu half‐cells, cycled for five cycles at 1 mA cm^−2^ and 1 mAh cm^−2^, were examined. As shown in Figure 2d, the Zn electrode cycled in 2 M ZnSO_4_ electrolyte exhibited a distinct S 2p peak centered around 170 eV, corresponding to the formation of Zn_4_SO_4_(OH)_6_.5H_2_O. Notably, this peak remained strong after an extensive 900‐second etching process of the electrode (20 keV, 1000+ Argon). In contrast, the Zn electrode cycled in 0.5 Cs_2_SnCl_6_ electrolyte initially displayed an S 2p peak, which completely dissipated after just 90 s of etching (Figure 2e). This rapid disappearance of the S signal indicates a significantly reduced incidence of side reactions on the Zn electrode in the presence of Cs_2_SnCl_6_. Furthermore, the consistent presence of the Sn 3d_5/2_ peak from the beginning strongly supports the formation of a Zn─Sn alloy. The XPS results provide further evidence of Zn─Sn alloy formation, as demonstrated by the presence of Sn signals on the electrodes after the stripping phase (Figure 2f), and their absence after the deposition phase (Figure S14, Supporting Information). This suggests that Sn actively participates in the Zn nucleation stage, contributing to the reduction of NOP and promoting more uniform Zn deposition.

### 2.3 Electrostatic Shield and pH Buffer Effects

The presence of Cs^+^ ions in the Cs_2_SnCl_6_‐modified also promotes compact and uniform Zn deposition. This improvement is largely attributed to the Self‐Healing Electrostatic Shield (SHES) mechanism, which facilitates dendrite‐free Zn deposition in the 0.5 Cs_2_SnCl_6_ electrolyte.^[^ ^38^ ^]^ During Zn deposition process, electric field fluctuations on the surface of the Zn anode can lead to the formation of protrusions. In the pristine electrolyte, these protrusions intensify local electric fields, causing Zn^2+^ ions to deposit preferentially at the tips of the protrusions rather than on the flatter regions of the Zn anode. This uneven deposition promotes Zn dendrite formation, ultimately causing battery short‐circuiting, as illustrated in Figure S15a (Supporting Information). In contrast, in the 0.5 Cs_2_SnCl_6_ electrolyte, Cs^+^ ions accumulate at the tips of the protrusions and form a stable electrostatic barrier due to their lower reduction potential compared to Zn^2+^ ions (Figure S15b, Supporting Information). This positively charged barrier repels Zn^2+^ ions from the protrusions, redirecting Zn deposition to adjacent regions of the anode and promoting a smoother deposition layer. Furthermore, XPS spectra (Figure S16, Supporting Information) revealed the absence of a Cs 3d signal, indicating that Cs^+^ ions remain in the electrolyte, rather than being deposited on the Zn anode surface.

The pH variations of the aqueous electrolytes for ZMBs can serve as an indicator of side reactions. It was observed that the pH values of the ZnSO_4_ electrolyte at the end of each discharge process significantly increased from 3.61 to 3.81 within 9600 s, indicating severe HER at the Zn interface (Figure 2g). In contrast, the pH values of the 0.5 Cs_2_SnCl_6_ electrolyte remained virtually unchanged over the same period, increasing only slightly from 2.65 to 2.68. A similar trend was observed at the end of each charge process (Figure 2h). Additionally, the degradation of the remaining Cs_2_SnCl_6_ additive can release protons, which can stabilize the local pH values of the aqueous electrolytes during cycling.^[^ ^39^ ^]^ As a result, the 0.5 Cs_2_SnCl_6_ electrolyte exhibited fewer side reactions, contributing to enhanced cycling stability.

**Figure 3:**
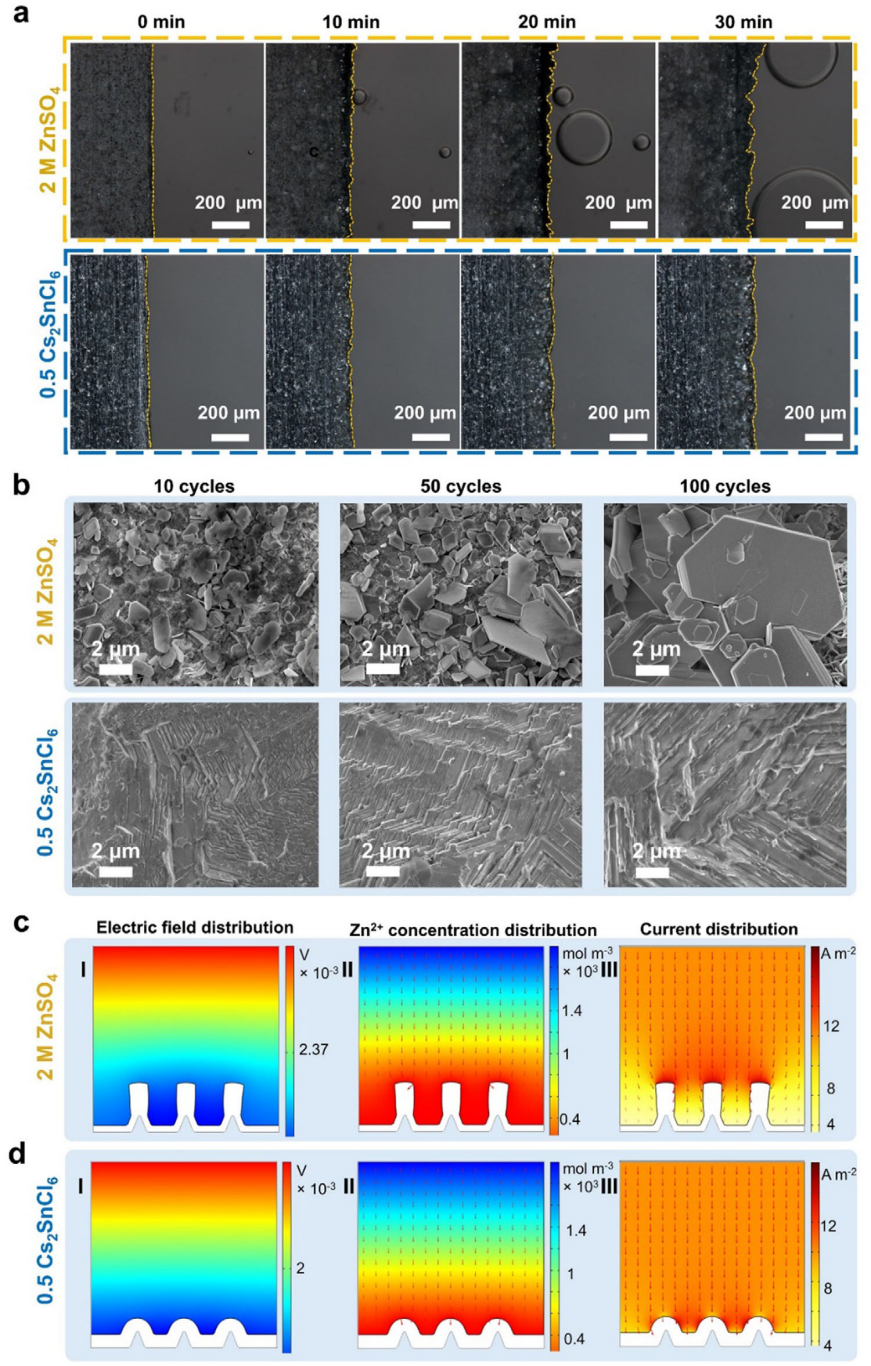
a) In situ optical microscopy observations of Zn^2+^ deposition process at 5 mA cm^−2^. b) The SEM images of Zn||Zn symmetric cells after 10, 50, and 100 cycles in 2 m ZnSO_4_ and 0.5 Cs_2_SnCl_6_ electrolytes. c) COMSOL simulation result of the electrode in 2 m ZnSO_4_ and d) in 0.5 Cs_2_SnCl_6_ electrolytes.

An in situ microscopy system was employed to monitor the morphological changes of the Zn anode in various electrolytes during the continuous plating. As shown in **Figure** **3**a, in the 2 m ZnSO_4_ electrolyte, numerous non‐uniform nucleation sites and small bubbles appeared on the electrode surface within 10 min of plating. After 30 min of deposition, severe dendrites were observed, accompanied by the appearance of large bubbles. In contrast, the electrodeposition process of Zn^2+^ ions in the 0.5 Cs_2_SnCl_6_ electrolyte was significantly more uniform, with no bubble formation detected throughout the deposition process.

The advantages of the 0.5 Cs_2_SnCl_6_ electrolyte in improving electrode morphology during cycling were further confirmed through SEM observation. As shown in Figure 3b, in the pristine ZnSO_4_ electrolyte, the Zn anode from the Zn||Zn symmetric cell developed a rough morphology covered by nanoflakes after just 10 cycles. After 100 cycles, these nanoflakes accumulated into micro‐sized hexagonal sheets, indicating severe side reactions and uneven Zn deposition. In contrast, in the 0.5 Cs_2_SnCl_6_ electrolyte, the Zn anode exhibited a flat and smooth surface without dendrite formation, even after 10, 50, and 100 cycles. Additionally, SEM images of the Cu electrode in the Zn||Cu half‐cell after 100 cycles, further demonstrated that Zn deposition in the 0.5 Cs_2_SnCl_6_ electrolyte was flatter and more densely packed, confirming its role in promoting more uniform and controlled Zn growth (Figure S17, Supporting Information).

From the previous study, the crystal planes, including Zn (110), Zn (101), and Zn (002), are prone to align the Zn growth direction.^[^ ^40^ ^]^ The (002) orientation, which is the preferred one, represents flatter and more uniform deposition. The ratio of Zn (002) to Zn (100) peaks (I_Zn_(002)/I_Zn_(100)) was used to check the dominant Zn growth direction on the electrode. After 10, 50, and 100 cycles, the ratio of I_Zn_(002)/I_Zn_(100) of the Zn electrode cycled in the 0.5 Cs_2_SnCl_6_ electrolyte (1.88, 2.392, and 2.827) was significantly higher than that of the Zn electrode cycled in the 2 m ZnSO_4_ electrolyte (0.343, 0.482, and 0.591) (Figure S18). This indicates a substantial preferential growth of the Zn (002) plane in the perovskite‐modified electrolyte, suggesting that Cs_2_SnCl_6_ influences the orientation of Zn deposition, leading to a more condensed and flat Zn layer.^[^ ^41^ ^]^ The observed enhancement in Zn (002) orientation may also be partially associated with the mildly acidic environment introduced by the Cs_2_SnCl_6_ additive. Trace protons at the Zn/electrolyte interface can modulate the electric double layer, reduce the nucleation barrier for (002) facets, and suppress growth along higher‐energy orientations such as (100), leading to smoother and more compact Zn deposition. The preferred Zn (002) growth is thus attributed to the combined influence of solvation structure optimization and interfacial proton regulation.^[^ ^42^, ^43^ ^]^

To comprehensively analyze the impact of Cs_2_SnCl_6_ additive on the electric field distributions, Zn^2+^ ion distributions, and current density distributions at the electrode/electrolyte interface, finite element modeling (FEM) simulations were performed using COMSOL. The deposition behaviors were systematically recorded from 0 s to 1000 s and is presented in Figure S19 (Supporting Information). In the absence of the Cs_2_SnCl_6_ additive, a highly concentrated electric field was observed around protrusion tips, promoting preferential Zn^2+^ growth at these sites and resulting in a more uneven electric field distribution (Figure 3c(I)). In contrast, the incorporation of Cs_2_SnCl_6_ significantly moderates the electric field inhomogeneities (Figure 3d(I)). This effect is primarily attributed to the dynamic electrostatic shielding by Cs^+^ ions, which effectively redistributes the Zn^2+^ ions away from the high electric field regions, encouraging deposition at adjacent, flatter sites with lower reactivity and reduced surface energy. This redistribution facilitates uniform charge dispersion, culminating in a potential compensation effect (Figures 3d(II)). In contrast, in pristine ZnSO₄ electrolyte, Zn^2+^ ions predominantly migrate under the influence of localized high electric fields, leading to preferential deposition in regions with high potential gradients. This deposition behavior often results in dendrite formation, as Zn^2+^ tends to accumulate at highly reactive sites with elevated surface energy (Figure 3 c(II)).

Furthermore, the simulation revealed an extremely high current density at Zn protuberances in the ZnSO₄ electrolyte, indicating localized 3D growth. This uneven distribution promotes sustained dendritic formation, as Zn^2+^ ions preferentially deposit at high‐current regions, exacerbating interfacial instability (Figure 3c(III)). However, in the 0.5 Cs_2_SnCl_6_ electrolyte, the presence of Cs^+^ ions fostered a more uniform Zn^2+^ ion distribution around Zn protuberances. This uniformity is particularly beneficial in mitigating Zn dendrite formation at protrusion tips (Figure 3d(III)). This comprehensive simulation underscores the pivotal role of Cs_2_SnCl_6_ in promoting uniform Zn deposition.

### 2.4 Electrostatic Shield and pH Buffer Effects

**Figure 4:**
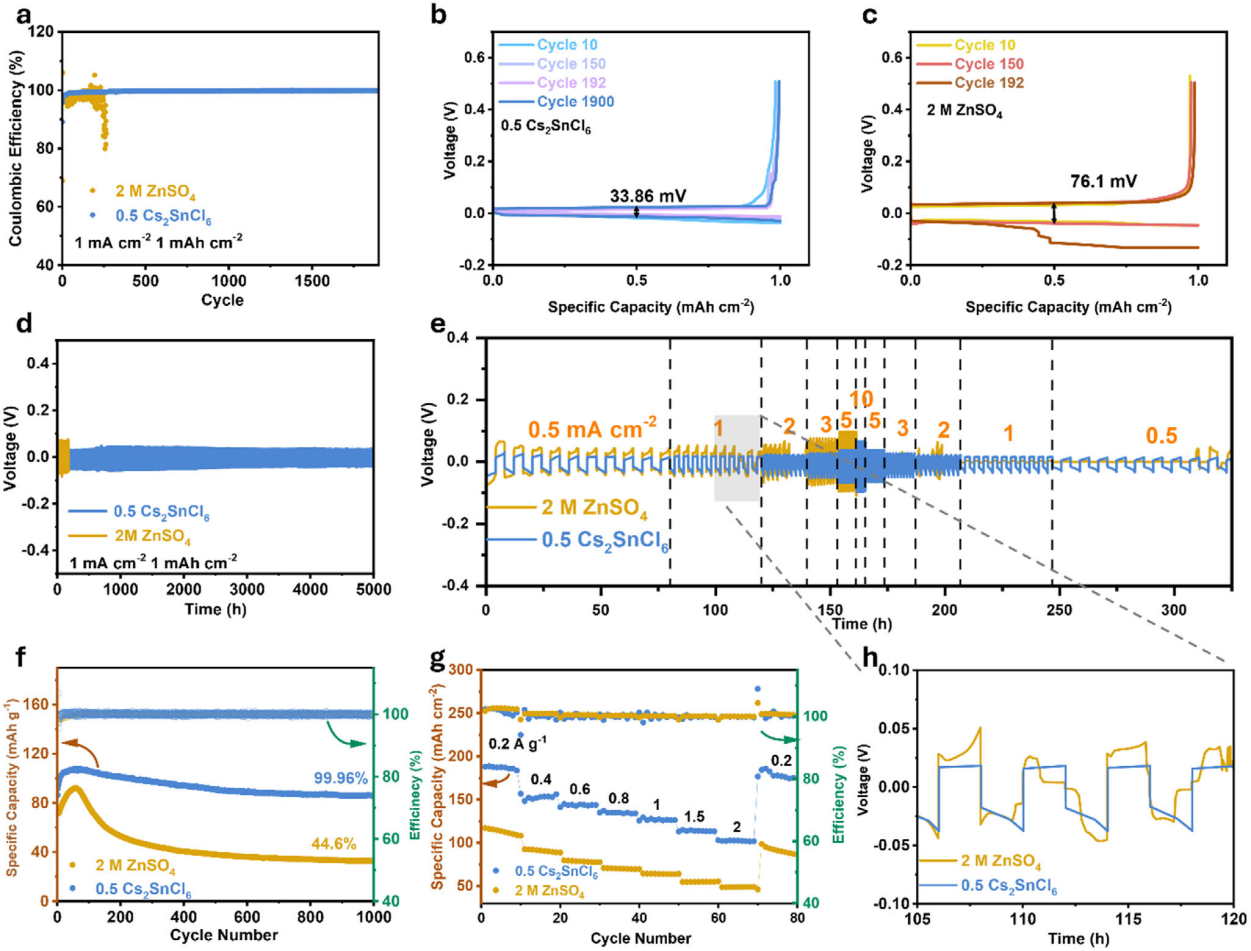
a) CE test of Zn||Cu half‐cells using ZnSO_4_ electrolyte and 0.5 Cs_2_SnCl_6_ electrolyte. The corresponding voltage profiles of Zn||Cu cell with b) 0.5 Cs_2_SnCl_6_ electrolyte, and c) 2 m ZnSO_4_ electrolyte. d) Cycling performance of Zn||Zn symmetric cells with ZnSO_4_ and 0.5 Cs_2_SnCl_6_ electrolyte at 1 mA cm^−2^, 1 mAh cm^−2^. e) Rate performance of Zn||Zn symmetric cells using ZnSO_4_ and 0.5 Cs_2_SnCl_6_ electrolyte. f) Cycling performance of Zn||V_2_O_5_ full cells in the ZnSO_4_ electrolyte and 0.5 Cs_2_SnC_6_ electrolyte at 1 A g^−1^. g) Rate performance of Zn||V_2_O_5_ full cells in different electrolytes. h) The corresponding voltage profiles of the symmetric cells in the rate performance test.

The electrochemical performance of repeated Zn plating/stripping was conducted in Zn||Cu half cells by measuring the Coulombic efficiencies (CEs). As shown in **Figure** **4**a, the Zn||Cu cell with 0.5 Cs_2_SnCl_6_ electrolyte achieved an average CE of 99.6% after 1900 cycles at 1 mA cm^−2^ and 1 mA h cm^−2^. In contrast, the CEs of the cell with the ZnSO_4_ electrolyte declined significantly after 193 cycles. The cells containing Cs_2_SnCl_6_ also exhibited improved cycle life at higher current density and area capacity (3 mA cm^−2^, 3 mAh cm^−2^) (Figure S20, Supporting Information). Furthermore, the cell with 0.5 Cs_2_SnCl_6_ electrolyte displayed an overpotential of 33.86 mV (Figure 4b), which was much lower than that of the cell with ZnSO_4_ electrolyte (76.1 mV) (Figure 4c).

The cycling performance of Zn anodes in different electrolytes was further conducted using Zn||Zn symmetric cells. The inclusion of Cs_2_SnCl_6_ significantly enhanced the cycling stability of the symmetric cells, achieving remarkable stability up to 5000 h at 1 mA cm^−2^ and 1 mA h cm^−2^ (Figure 4d), 2000 h at 3 mA cm^−2^ and 3 mA h cm^−2^, 800 h at 5 mA cm^−2^ and 5 mA h cm^−2^, and 450 h at 10 mA cm^−2^ and 5 mA h cm^−2^ (Figure S21, Supporting Information). The effect of different Cs_2_SnCl_6_ concentrations on cycling performance was also analyzed (Figure S22, Supporting Information), with the 0.5 Cs_2_SnCl_6_ electrolyte demonstrating the best cycling performance. The relatively lower improvement observed in the 2 Cs_2_SnCl_6_ electrolyte may be attributed to its low pH, which could accelerate anode corrosion (Figure S23, Supporting Information). In addition, the rate performance of symmetric cells with and without Cs_2_SnCl_6_ was assessed across a range of current densities from 0.5 to 10 mA cm^−2^ (Figure 4e). The overpotentials of the cell containing 0.5 Cs_2_SnCl_6_ electrolyte were significantly lower compared to the cell with ZnSO_4_ electrolyte throughout the cycling process. Moreover, the symmetric cell using 2 M ZnSO_4_ electrolyte experienced a short circuit after only 110 h of operation (Figure 4h). These results demonstrate the substantial impact of Cs_2_SnCl_6_ on improving the durability and efficiency of Zn anodes. The effect of directly adding different hydrolysis products of Cs_2_SnCl_6_ to the electrolyte on battery performance was also investigated (Figures S24 and S25, Supporting Information). Compared to the symmetric cell with ZnSO_4_ electrolyte, adding SnCl_4_·5H_2_O or CsCl at various concentrations did not significantly improve the battery's cycle life.

To evaluate the impact of the 0.5 Cs_2_SnCl_6_ electrolyte on the electrochemical performance of full cells, commercial V_2_O_5_ was used as the cathode material, paired with a Zn metal anode. The XRD and SEM results of the V_2_O_5_ powder are shown in Figure S26 (Supporting Information), confirming its phase purity and large micron‐sized particle morphology. At a current density of 1 A g^−1^, the full cell exhibited enhanced cycling stability, reaching a peak discharge capacity of 107 mA h g^−1^ and retaining 99.96% of the initial capacity after 1000 cycles (Figure 4f). As shown in Figure 4g, the full cell with the Cs_2_SnCl_6_ additive demonstrated superior rate performance compared to the cell without the additive. The extended galvanostatic charge‐discharge curves under long‐term cycling further confirm this enhancement (Figure S27, Supporting Information). These results highlight the significant role of the 0.5 Cs_2_SnCl_6_ electrolyte in improving both capacity and long‐term cycling stability of ZMBs.

## 3 Conclusion

In summary, we introduce Cs_2_SnCl_6_ as a multi‐functional electrolyte additive for aqueous Zn metal batteries, significantly enhancing their cycling stability and rate performance. Through a combination of experimental studies and theoretical calculations, we demonstrate that the metal halide perovskite‐modified electrolyte (0.5 Cs_2_SnCl_6_ in 2 m ZnSO_4_ solution) effectively regulates the solvation structure of Zn^2+^ ions, which assists nucleation process, inhibits Zn dendrite formation and side reactions, and stabilizes pH of electrolyte during cycling. Raman spectroscopy and NMR data reveal that the Cl^−^ ions released from Cs_2_SnCl_6_ preferentially interact with Zn^2+^ ions over water molecules, optimizing the solvation structure of Zn^2+^ ions and thereby mitigating side reactions caused by solvated water molecules at the Zn anode. The dissolved Sn^4+^ ions aid the nucleation process by forming a zincophilic Zn‐Sn alloy in the initial nucleation stage, which promotes uniform Zn plating and reduces side reactions. Additionally, the Cs^+^ ions from the Cs_2_SnCl_6_ additive follow the SHES mechanism, effectively preventing Zn dendrite growth. Furthermore, the partial hydrolysis of Cs_2_SnCl_6_ acts as a pH buffer, thereby significantly mitigating pH fluctuations during cycling. As a result, the electrolyte with the Cs_2_SnCl_6_ additive endows the Zn||Zn symmetric cells with exceptional long‐term cycling stability, achieving 5000‐hour cycle life at 1 mA cm^−2^ and 1 mAh cm^−2^. Moreover, the Zn||V_2_O_5_ full cell with 0.5 Cs_2_SnCl_6_ electrolyte delivered exceptional cycling stability, retaining 99.96% of the initial capacity after 1000 cycles. This work presents a simple and effective strategy for enhancing the cycle life of Zn metal anodes, paving the way for the practical development of Zn metal batteries with improved stability and efficiency.^[^ ^44^, ^45^, ^46^, ^47^, ^48^ ^]^

## Conflict of Interest

The authors declare no conflict of interest.

## Supporting information



Supporting Information

## Data Availability

The data that support the findings of this study are available from the corresponding author upon reasonable request.
