# Supplementary material for: Multifunctional Metal Halide Perovskite‐Modified Aqueous Electrolytes for Zinc Metal Batteries
Source: Adv Sci (Weinh). 2025 Jul 28;12(38):e09417. doi: 10.1002/advs.202509417 (PMC12520530; doi:10.1002/advs.202509417)
Supplement: Supplementary file 1 — Supporting Information [file ADVS-12-e09417-s001.docx]

Supporting Information

Multifunctional Metal Halide Perovskite-Modified Aqueous Electrolytes for Zinc Metal Batteries

Tong Yang, Jiahui Lu, Minh Tam Hoang, Yang Yang, Shijian Wang, Yong Chen, Yongyue Yu, Yan Wang, Timing Fang, Bing Sun*, Guoxiu Wang*, Hongxia Wang*

T. Yang, Dr M. Hoang, Dr Y. Yang, Y. Yu, Prof. H. Wang

School of Chemistry and Physics, Queensland University of Technology, 2 George St, Brisbane City QLD 4000, Australia
E-mail: [hx.wang@qut.edu.au](mailto:hx.wang@qut.edu.au).

J. Lu, Dr S. Wang, Y. Chen, Dr B. Sun, Prof. G. Wang
School of Mathematical and Physical Science, University of Technology Sydney, 15 Broadway, Ultimo NSW 2007, Australia

E-mail: [bing.sun@uts.edu.au](mailto:bing.sun@uts.edu.au), [Guoxiu.Wang@uts.edu.au](mailto:Guoxiu.Wang@uts.edu.au).

Y. Wang, T. Fang

College of Chemistry and Chemical Engineering, Qingdao University, Qingdao, Shandong 266071, P. R. China

Experiment Procedures

Chemicals and Materials.

Cesium chloride (CsCl, 99.9%, Sigma-Aldrich), tin(IV) Chloride pentahydrate (SnCl_4_.5H_2_O, 98%, Sigma-Aldrich), methyl alcohol (≥99.8% ACS reagent, Sigma-Aldrich), zinc sulfate heptahydrate (ZnSO_4_·7H_2_O, ACS reagent, 99%, Sigma-Aldrich), vanadium(V) oxide (V_2_O_5_, ≥98%, Sigma-Aldrich), Poly(vinylidene fluoride) (PVDF, average Mw ~534,000 by GPC, powder), 1-Methyl-2-pyrrolidinone (NMP, anhydrous, 99.5%, Sigma-Aldrich), carbon black (Super P, IMERYS), copper foil, zinc foil, stainless steel foil.

Synthesis of Cs_2_SnCl_6_ perovskite:

First, 1.0 mmol of CsCl was dissolved in 20 mL of methanol under constant magnetic stirring for 1 hour. Separately, 0.5 mmol of SnCl_4_·5H_2_O was dissolved in 5 mL of methanol with continuous stirring for 30 minutes. The SnCl_4_·5H_2_O solution was then added to the CsCl solution, and the mixture was stirred vigorously for an additional 30 minutes. Afterward, the solution was centrifuged at 8000 rpm for 5 minutes to precipitate the Cs_2_SnCl_6_ particles. The collected precipitate was dried in a vacuum oven at 80°C overnight to obtain the final Cs₂SnCl₆ product.

Preparation of electrolytes:

Deionized water was purged with nitrogen gas (N₂) to eliminate dissolved oxygen. Subsequently, a 2 M ZnSO_4_ electrolyte was prepared using zinc sulfate heptahydrate (ZnSO_4_·7H_2_O) and the pre-purged deionized water. Various concentrations of Cs_2_SnCl_6_ powder were then incorporated into the 2 M ZnSO_4_ electrolyte to prepare Cs_2_SnCl_6_-dispersed electrolytes. The hybrid electrolyte was subsequently sonicated for one hour and allowed to equilibrate at room temperature for 12 hours. The Cs_2_SnCl_6_ well-dispersed electrolyte will be collected as the electrolyte.

Cathode preparation:

50 mg PVDF was added to 2 ml NMP and stirring for at least 4 hours until PVDF is completely dissolved. Vanadium (V) oxide was mixed with super P and grind for 30 minutes. PVDF/NMP solution was added to V_2_O_5_/Super P mixed powder to prepared cathode ink. The weight ratio of V_2_O_5_, PVDF and Super P is 8:1:1. The produced slurry was coated on stainless steel with doctor blade. The coated stainless steel was dried at 80 °C overnight in the vacuum oven. The dried coated stainless steel was punched into round pieces of 12 mm diameter. The mass loading of the V_2_O_5_ electrode for the full cell testing was about 1 mg cm^-2^.

**Material Characterization:**

The morphologies of Zn anode materials were evaluated by Scanning electron microscopy (Zeiss Supra 55VP SEM). Crystalline structures of all of the Zn electrode, Cu electrode and perovskite were analysed by X-ray diffraction (XRD, Rigaku Smart Lab XRD). Raman spectra were collected on the Raman spectrometer (Renishaw Qontor Raman microscope) with an excitation of 532 nm. Surface composition states were analyzed on X-ray photoelectron spectrometer (Kratos Axis Supra) and the argon gas cluster ion source was used to etching the Zn electrode surface. The in-situ microscope images of the transparent cells were obtained on an optical microscope (Leica M125 Zoom Stereo Microscope). Zeta potential measurements were conducted using a Malvern Zetasizer Nano ZS, and nuclear magnetic resonance (NMR) spectra were acquired on a Bruker Avance IIIHD 600 MHz spectrometer.

**Electrochemical Measurement:**

CR2032-Type coin cells were used to assemble Zn metal batteries for electrochemical performance evaluation. Glass microfiber filters were used as the separator. The galvanostatic discharge/charge profiles for Zn||Zn symmetric cell, Zn||V_2_O_5_ full cell and Zn||Cu half-cell were collected to evaluate the cycling performance using Neware multichannel galvanostatic. Linear sweep voltammetry (LSV) were collected in a three-electrode configuration (Stainless stell as working electrode and counter electrode, Ag/AgCl as the reference electrode) at a scan rate of 1.0 mV s^-1^. Tafel plots were measured by scanning between -0.5 V and -1.5 V at 2 mV s^-1^ with Zn plate as the working electrode, platinum foil as the counter electrode and Ag/AgCl as the reference electrode. The current-time transient curves were collected by chronoamperometry (CA) measurement at a fixed overpotential of 20 mV. The Electrochemical impedance spectroscopy (EIS), Nucleation overpotential (NOP) LSV, Tafel plot, pH fluctuation during cycling and CA are all carried out on a VMP-300 Potentiostat.

The Zn^2+^ transference number is determined by testing Zn||Zn symmetric cells with various electrolytes, combining EIS measurements before and after performing a chronoamperometry (CA) test. It is calculated using the following equation:

$$t_{{Zn}^{2+}}=\frac{I_{s}(\Delta V-I_{0}R_{0})}{I_{0}(\Delta V-I_{s}R_{s})}$$

Where I_0_ and R_0_ represent the initial current and resistance, respectively. ΔV refers to the applied voltage polarization, while I_s_ and R_s_ denote the steady-state current and resistance.

To determine the desolvation energy (E_a_) associated with the Zn deposition process across various electrolytes, symmetric Zn||Zn cells were utilized to conduct EIS (Electrochemical Impedance Spectroscopy) at varying temperatures. The E_a_ values were calculated from the Arrhenius equation.

$$\frac{1}{R_{ct}}=Aexp(-\frac{E_{a}}{RT})$$

Where R_ct_ is the charge transfer resistance obtained from the EIS spectra, R is the gas constant, and T is the thermos dynamic temperature.

The ionic conductivities of 2 M ZnSO_4_ and 0.5 Cs_2_SnCl_6_ electrolyte were evaluated based on the following equation:

$$\sigma=\frac{L}{R\times S}$$

Where R represents the resistance based on the EIS measurement of Zn||Zn symmetric cells filled with different electrolytes, L refers to the thickness of the separator, and S is the contact area.

**COMSOL Simulation:**

The concentration of different ions in the electrolyte is calculated according to the pH change and degradation equation of Cs_2_SnCl_6_. After added 0.5 w% of Cs_2_SnCl_6_, the pH changes from 4.15 to 2.58. The main reaction in the electrolyte can be described with following equation.

Cs_2_SnCl_6_ + 4H_2_O →2Cs^+^ + Sn(OH)_4_ + 4 H^+^ + 6 Cl^-^ Eq 4

Following the principle of acid dissociation and the relationship between pH and H^+^, the concentration shift of H^+^ in the electrolyte can be determined, followed by the concentrations of Cs^+^, Cl^-^ and Sn(OH)_4_. In this low pH environment, the Sn(OH)_4_ is stable and assists the Zn-Sn alloy synthesis. Cs^+^ assist the SHSE process and Cl^-^ engaged in the solvent structure modification.

**Molecular Dynamics Simulations:**

Molecular dynamics (MD) simulations were conducted to study the solvation structures and scrutinize the free water content of different aqueous electrolytes. The MD simulations were run by using LAMMPS ^[40]^. The systems are setup initially by using PACKMOL and Moltemplate ^[44]^. The properties of H_2_O are assessed with SPC/E parameters. The force–fields parameters and partial charges of Zn^2+^, SO_4_^2-^, Cl^-^, Cs^+^, and Sn^4+^ are taken from previous publications ^[45-48]^.

Supporting Figures


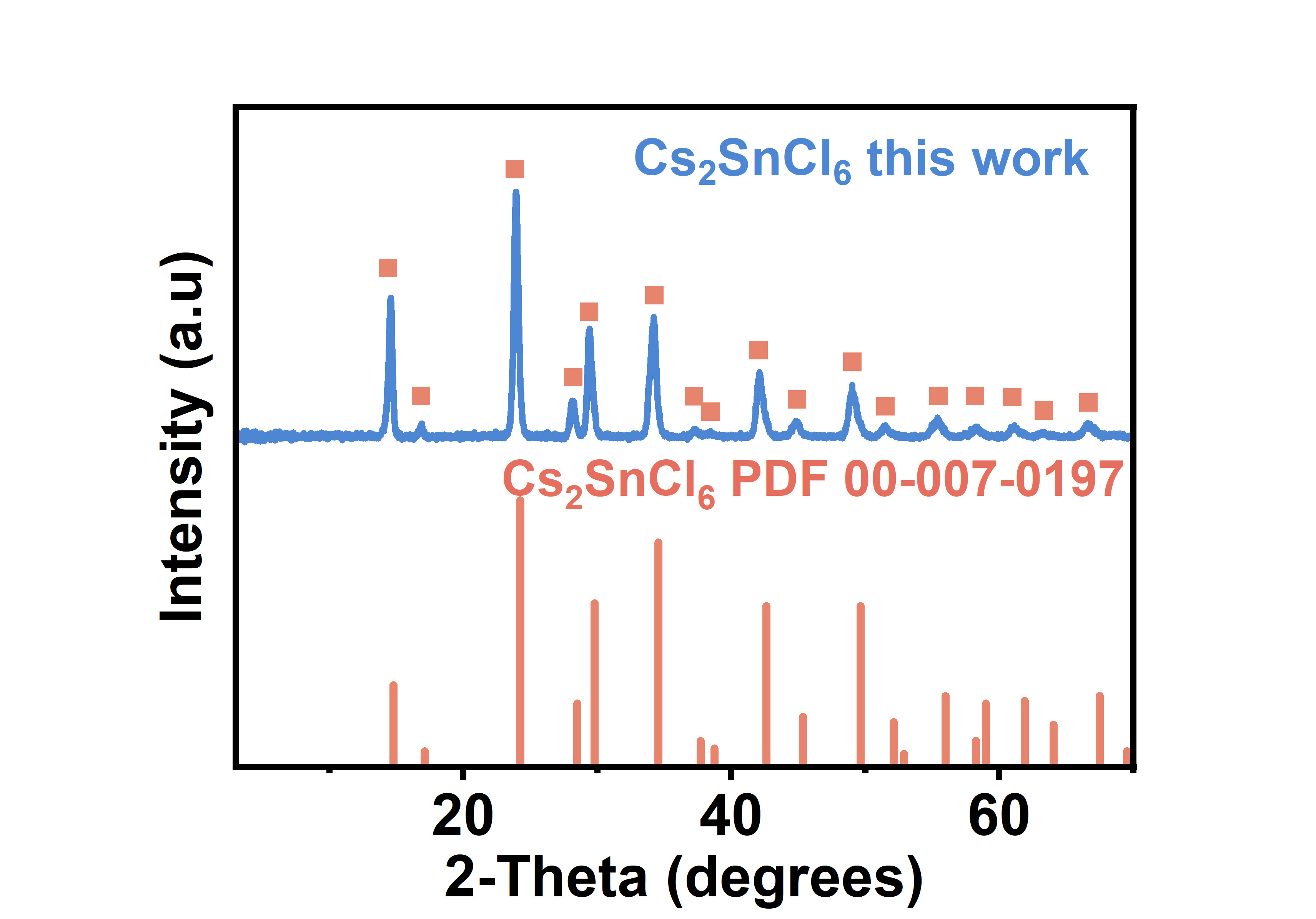


**Figure S1.** The XRD pattern of as-prepared Cs_2_SnCl_6_ powder.


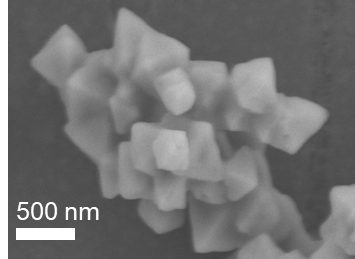


**Figure S2.** The SEM image of as-prepared Cs_2_SnCl_6_ powder.


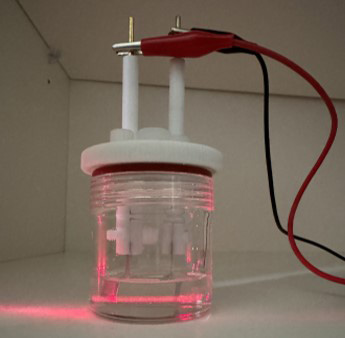


**Figure S3.** Tyndall effect of the 0.5 Cs_2_SnCl_6_ electrolyte during the electrochemical test.


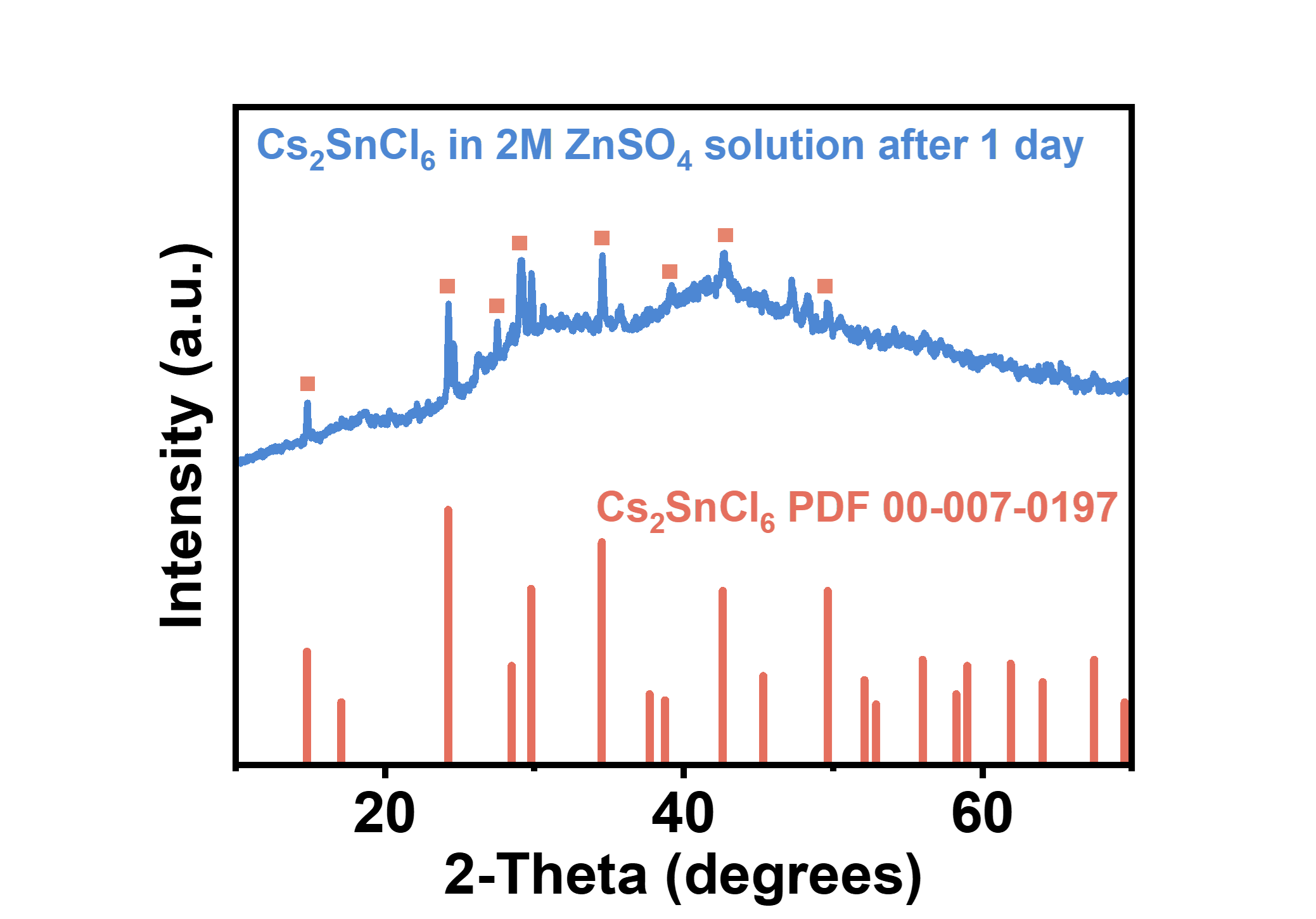


**Figure S4.** The XRD pattern of the centrifuged precipitate from 2 M ZnSO_4_ electrolyte containing Cs_2_SnCl_6_.


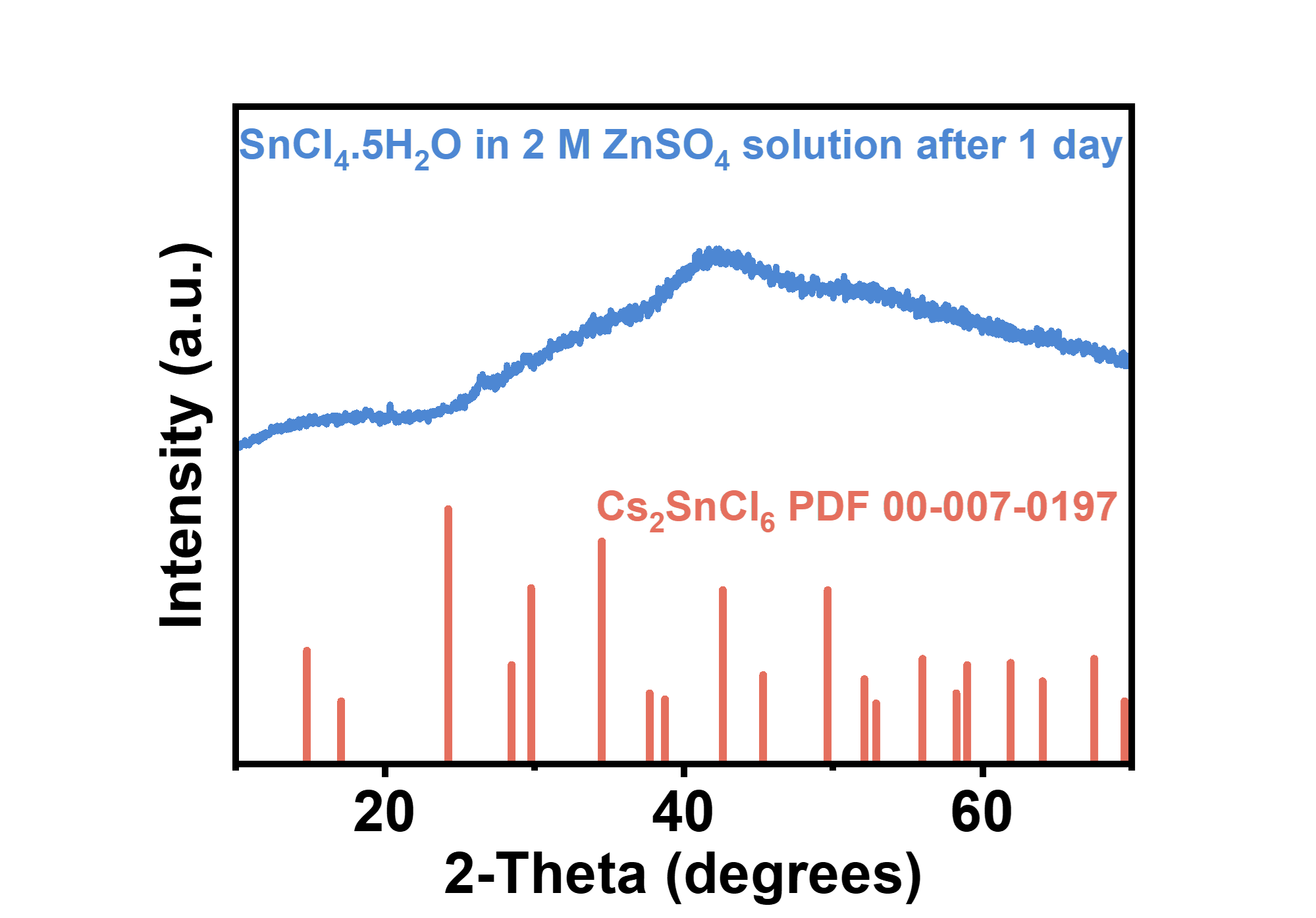


**Figure S5.** The XRD pattern of the centrifuged precipitate from 2 M ZnSO_4_ electrolyte containing SnCl_4_·5H_2_O.


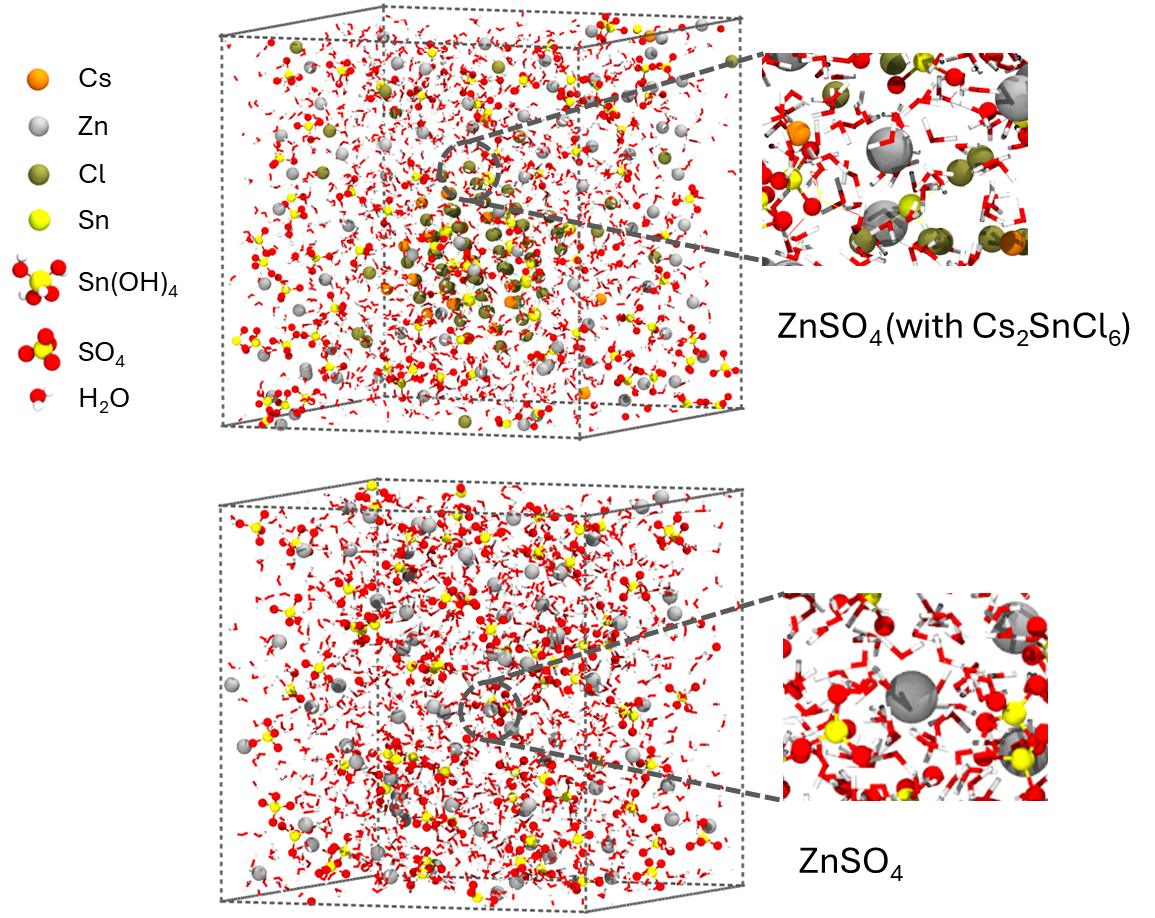


**Figure S6**. Molecular dynamics (MD) Simulation snapshots of the Cs_2_SnCl_6_ electrolyte and the local solvation structure of hydrated Zn^2+^ ions.


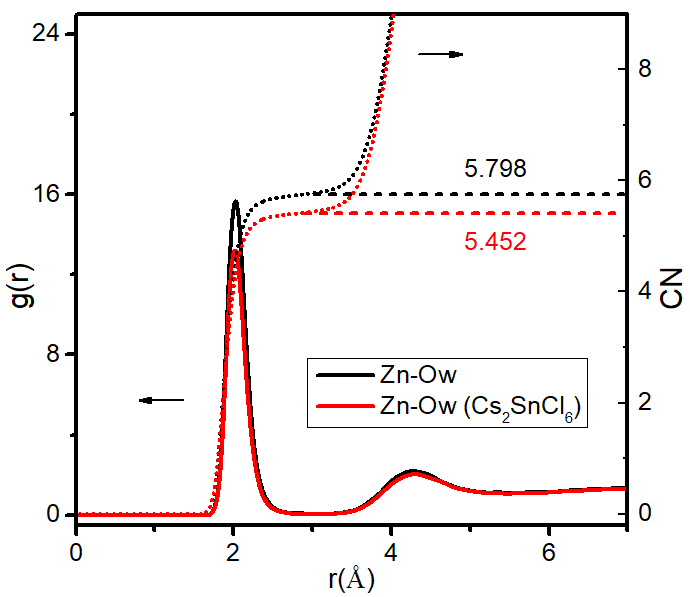


**Figure S7** The radial distribution function (RDF) for Zn-Ow from MD simulations in the 2 M ZnSO_4_ electrolytes and 0.5 Cs_2_SnCl_6_ electrolyte.


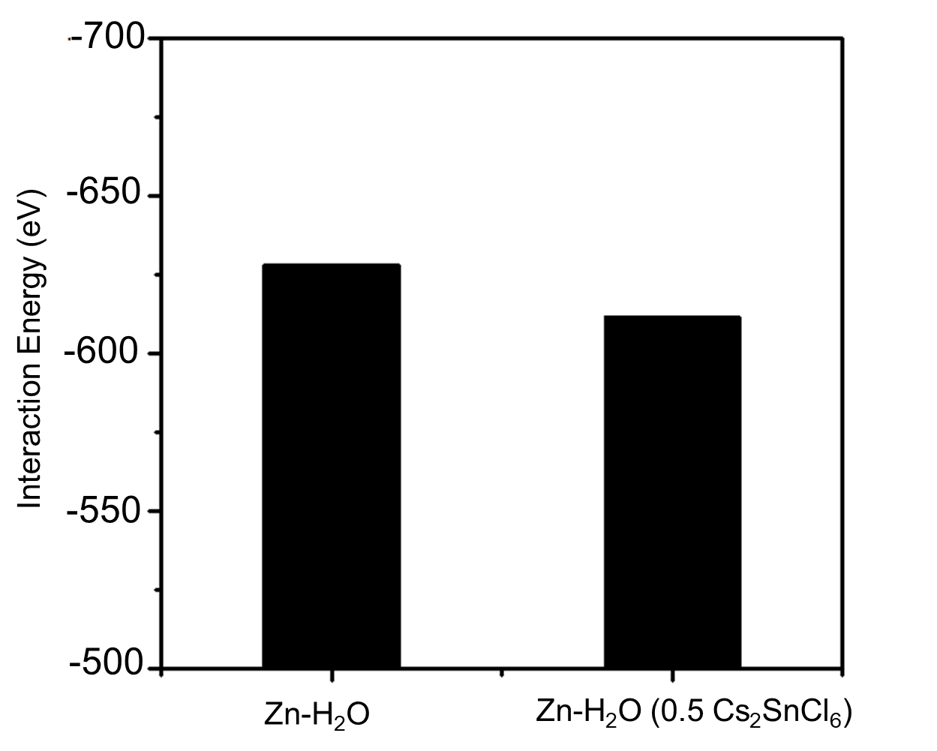


**Figure S8.** Interaction energy of 78 Zn-H_2_O and 78 Zn-H_2_O in 0.5 Cs_2_SnCl_6_ electrolyte.


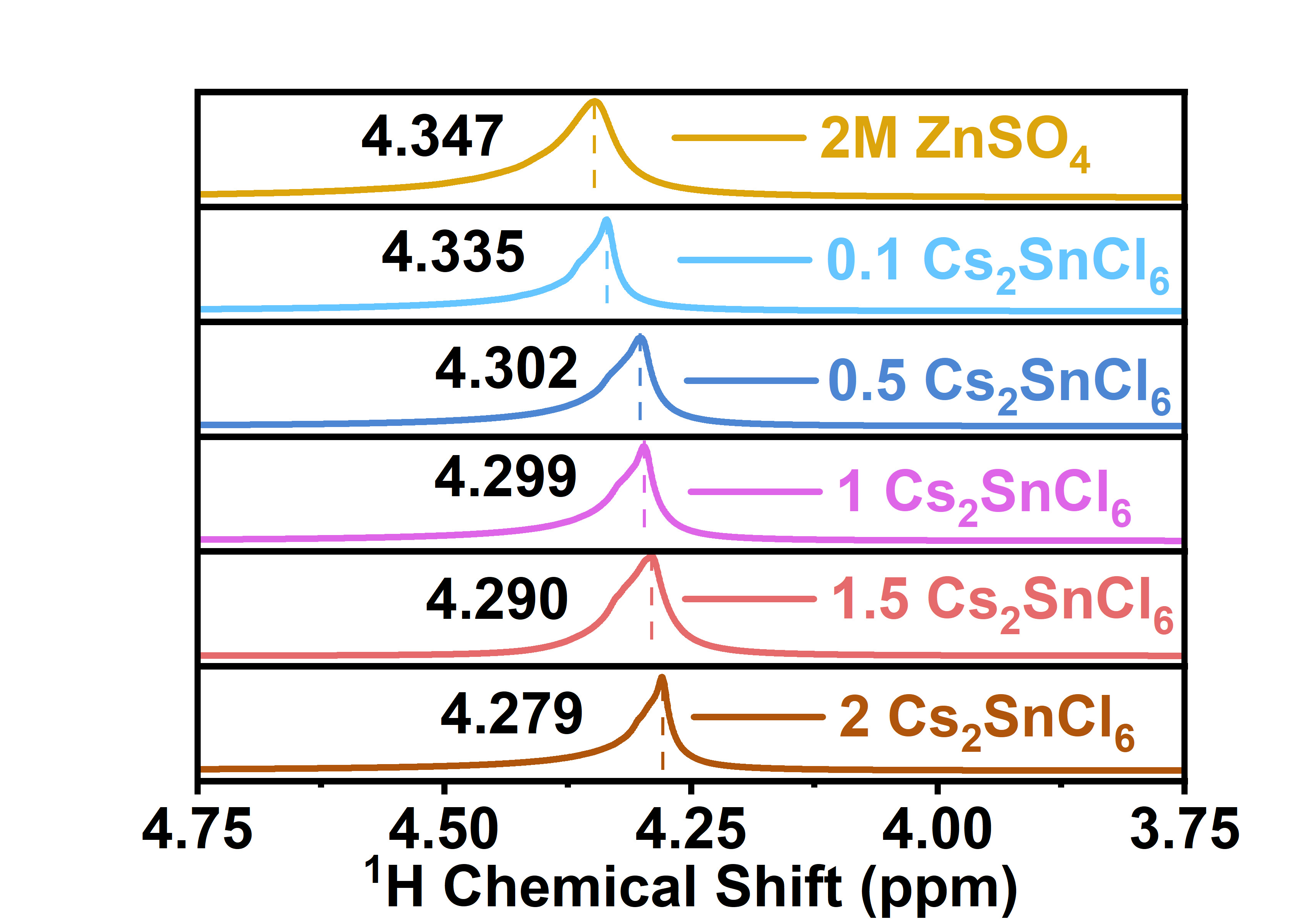


**Figure S9.** ^1^H NMR spectrum of ZnSO_4_ electrolyte and different Cs_2_SnCl_6_ modified ZnSO_4_ electrolytes.


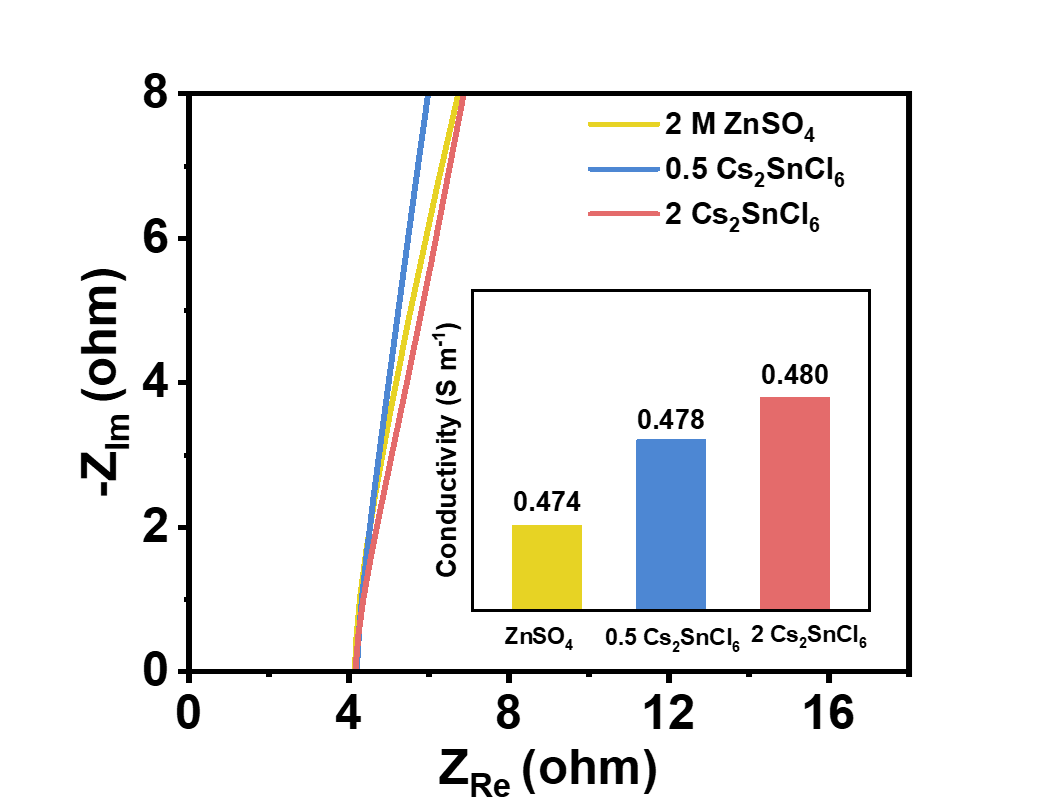


**Figure S10.** Impedance spectra and the corresponding ionic conductivities of 2 M ZnSO_4_ and 0.5 Cs_2_SnCl_6_ electrolytes. The addition of Cs₂SnCl₆ had a negligible effect on the ionic conductivity of the aqueous electrolytes.


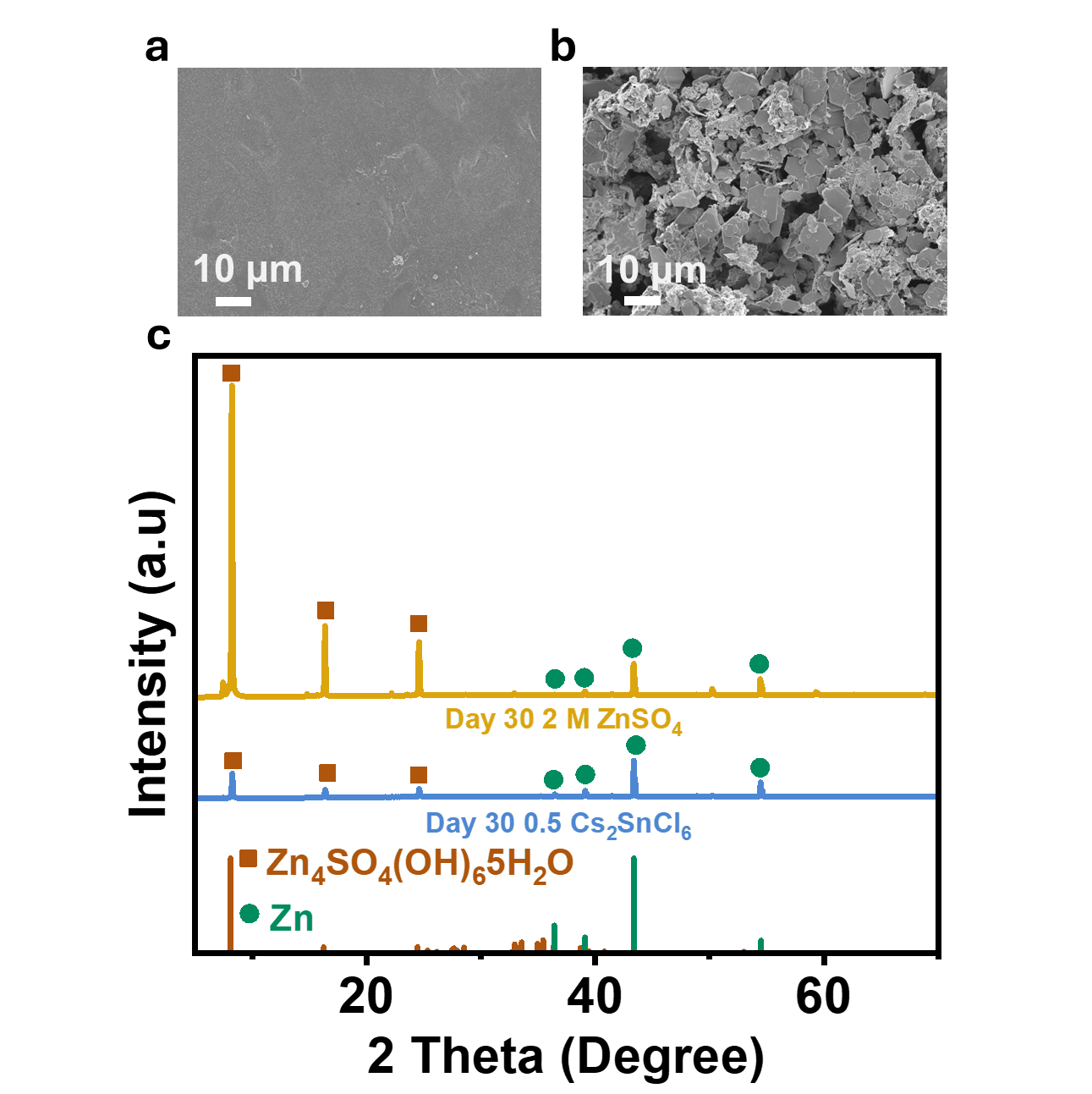


**Figure S11.** SEM images of the Zn electrodes after immersing in (a) 0.5 Cs_2_SnCl_6_ electrolyte, (b) and ZnSO_4_ electrolyte for 30 days, and (c) XRD pattern of the Zn electrodes after immersing test.


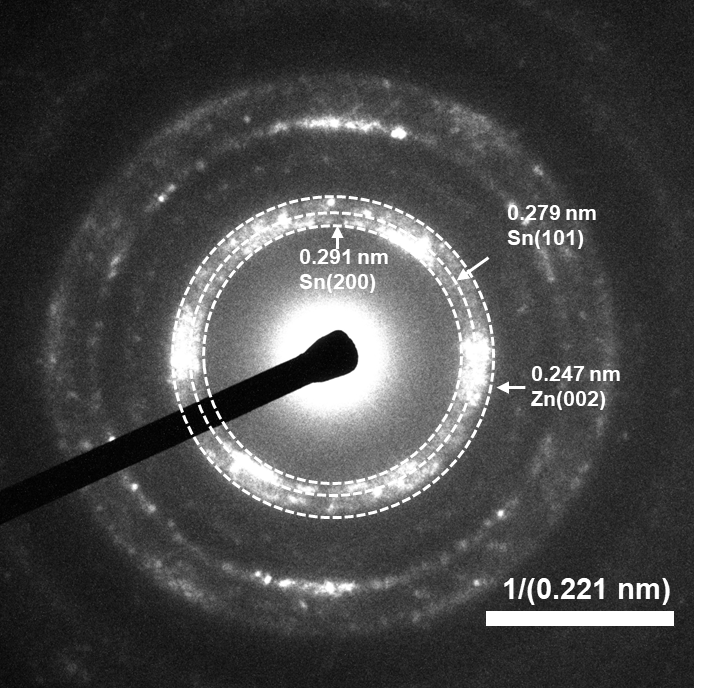


**Figure S12.** The selected area electron diffraction pattern of the Zn electrode after 5 cycles in the 0.5 Cs_2_SnCl_6_ electrolyte at the current density of 0.1 mA cm^-2^ with the capacity limitation of 0.1 mAh cm^-2^.


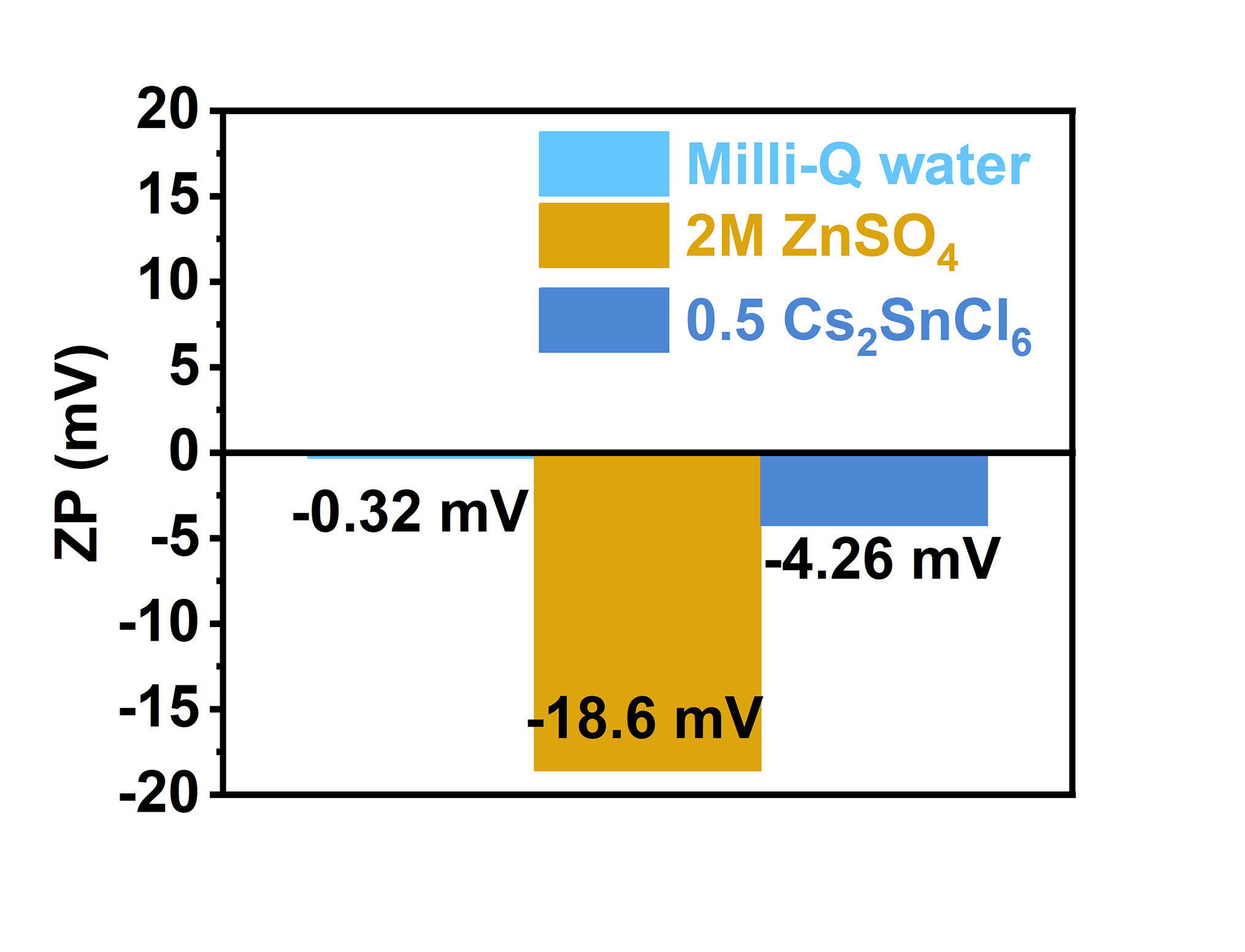


**Figure S13.** Zeta-potential of the Zn electrodes in the 2 M ZnSO_4_ electrolyte and 0.5 Cs_2_SnCl_6_ electrolyte.


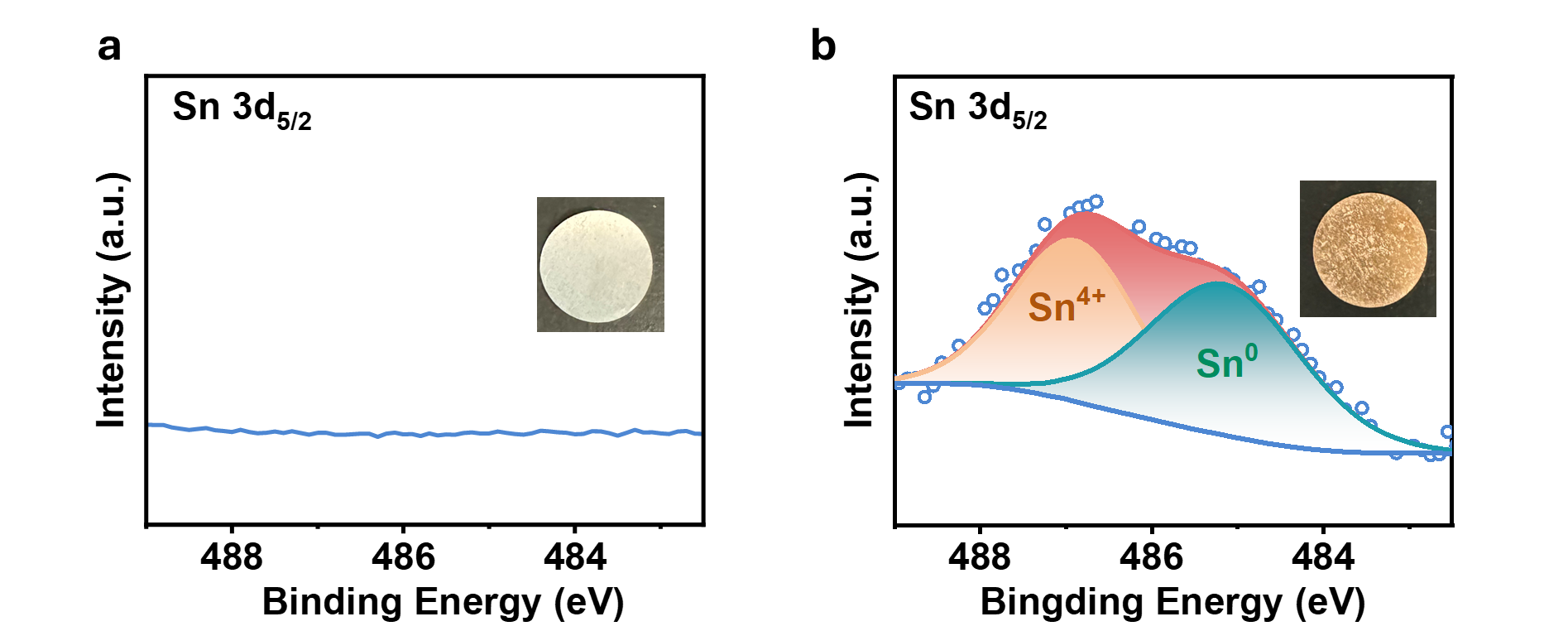


**Figure S14.** XPS spectra of Cu electrodes of the Zn||Cu half-cells cycled in the 0.5 Cs_2_SnCl_6_ (a) after 6th plating process, and (b) after 5 cycles at the stripping stage. Insets are the photos of the Cu electrodes.


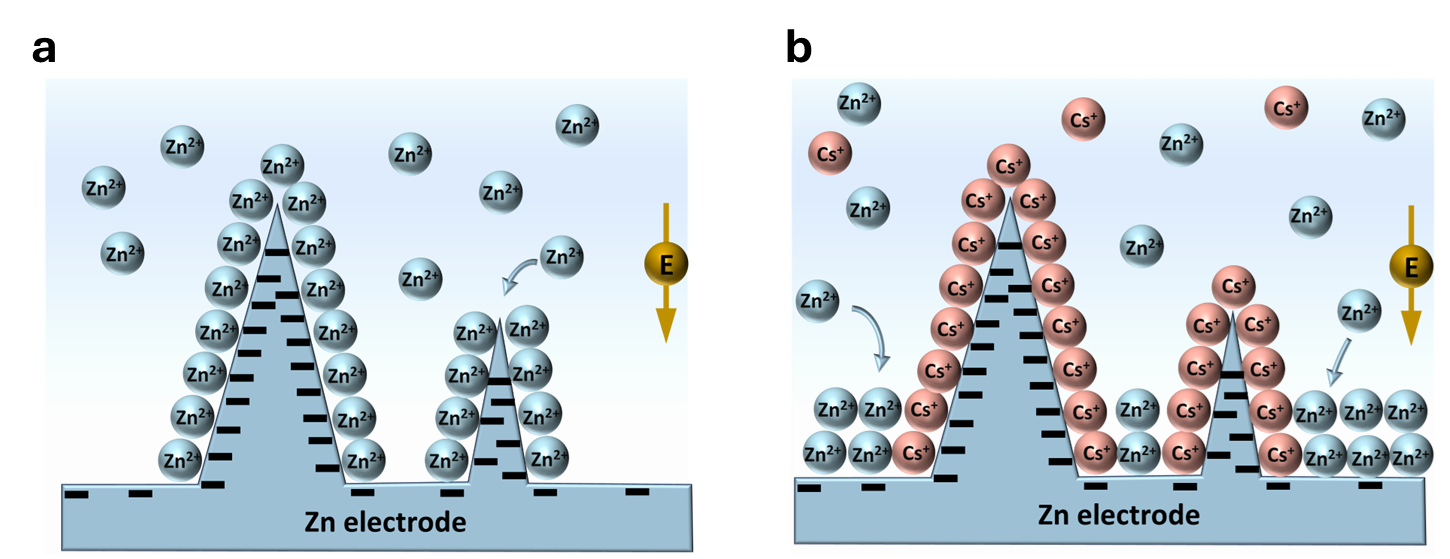


**Figure S15** Schematic illustrations of Zn^2+^ deposition process with and without Self-Healing Electrostatic Shield (SHES) effect in (a) 2 M ZnSO_4_ electrolyte, and (b) Cs_2_SnCl_6_ added electrolyte.


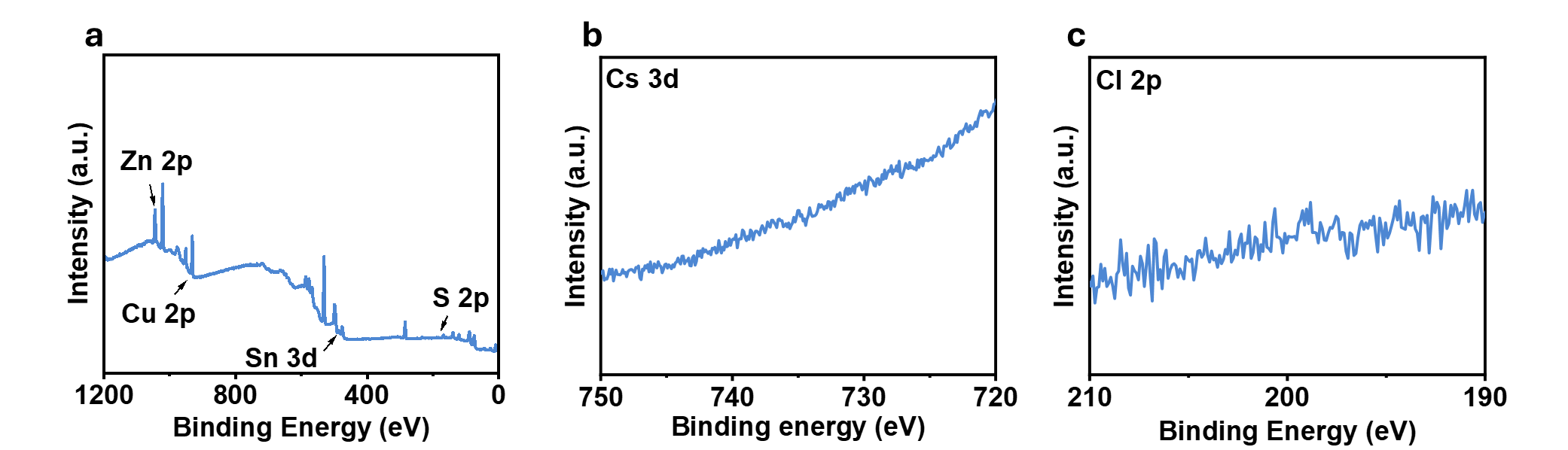


**Figure S16** XPS spectra of a Cu electrode from a Zn||Cu half-cell after 5 cycles in 0.5 M Cs_2_SnCl_6_ electrolyte at 1 mA cm^-2^ and 1 mAh cm^-2^. (a) wide scan, (b) Cs 3d region, and (c) Cl 2p region.


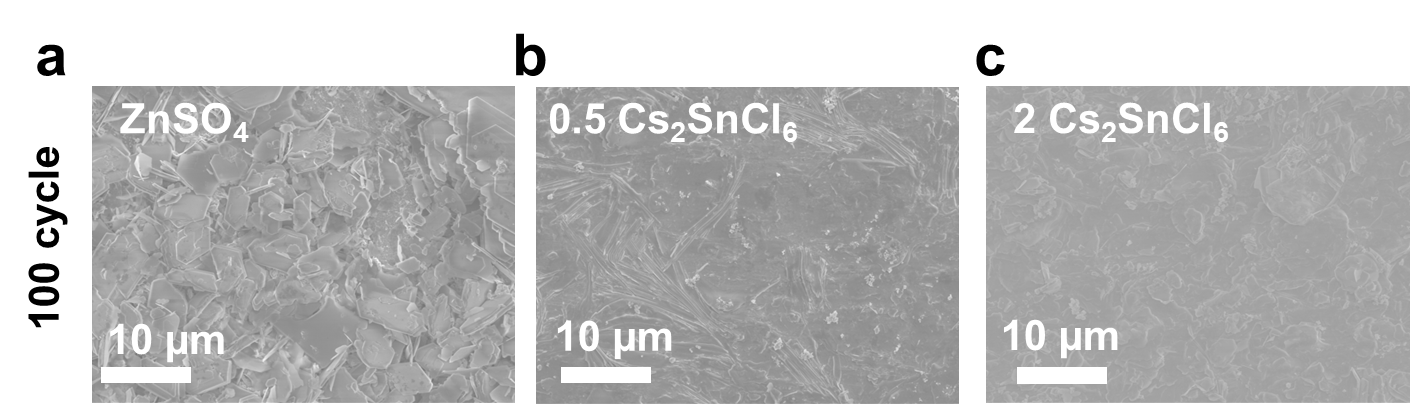


**Figure S17**. SEM images of the Zn deposited on Cu electrodes from Zn||Cu half cell after 100 cycles at 1 mA cm^-2^ and 1 mAh cm^-2^ in (a) ZnSO_4_ electrolyte, (b) 0.5 M Cs_2_SnCl_6_ electrolyte, and (c) 2 M Cs_2_SnCl_6_ electrolyte.


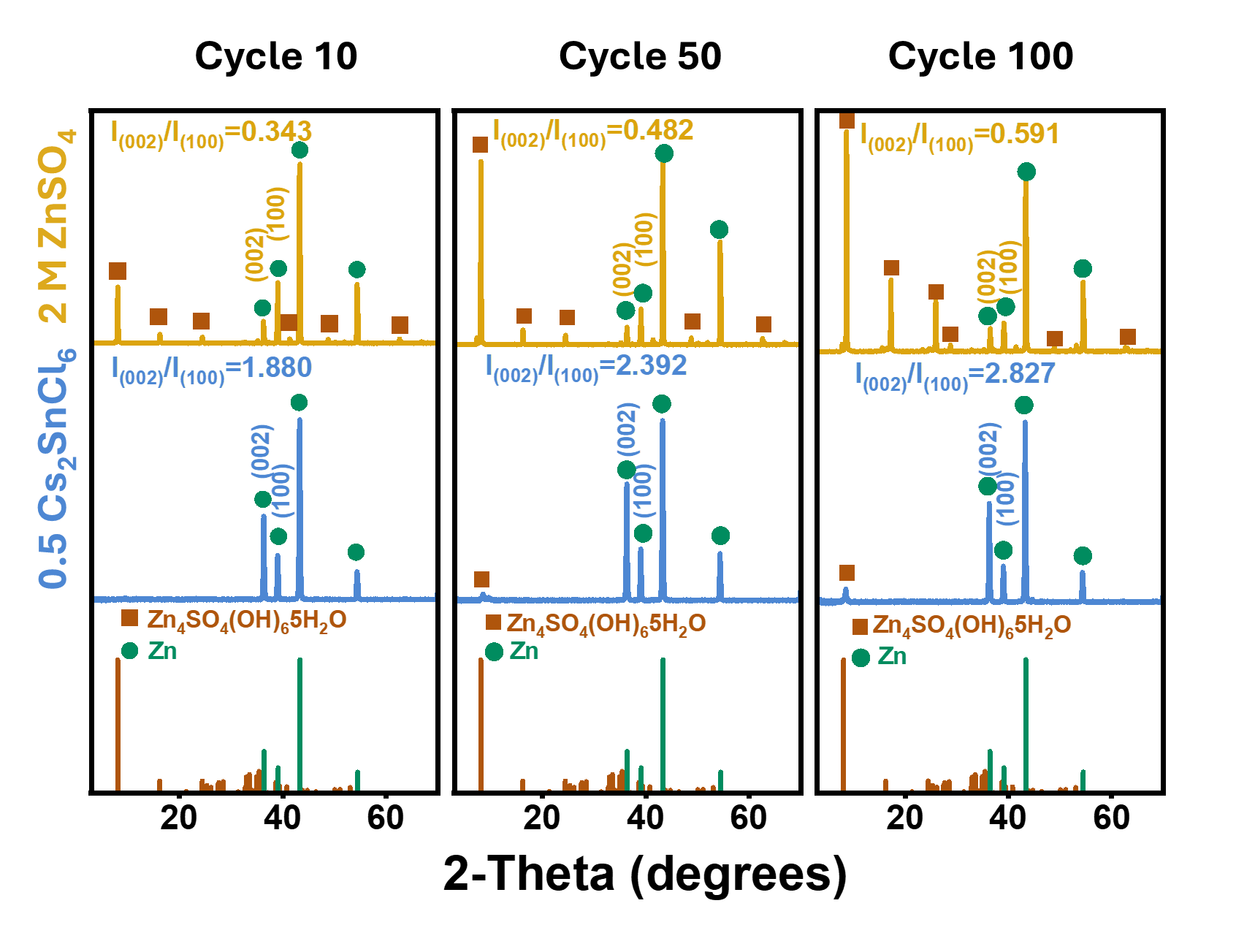


**Figure S18.** XRD patterns of the Zn electrodes of Zn||Zn symmetric cells with different electrolytes after different cycles.


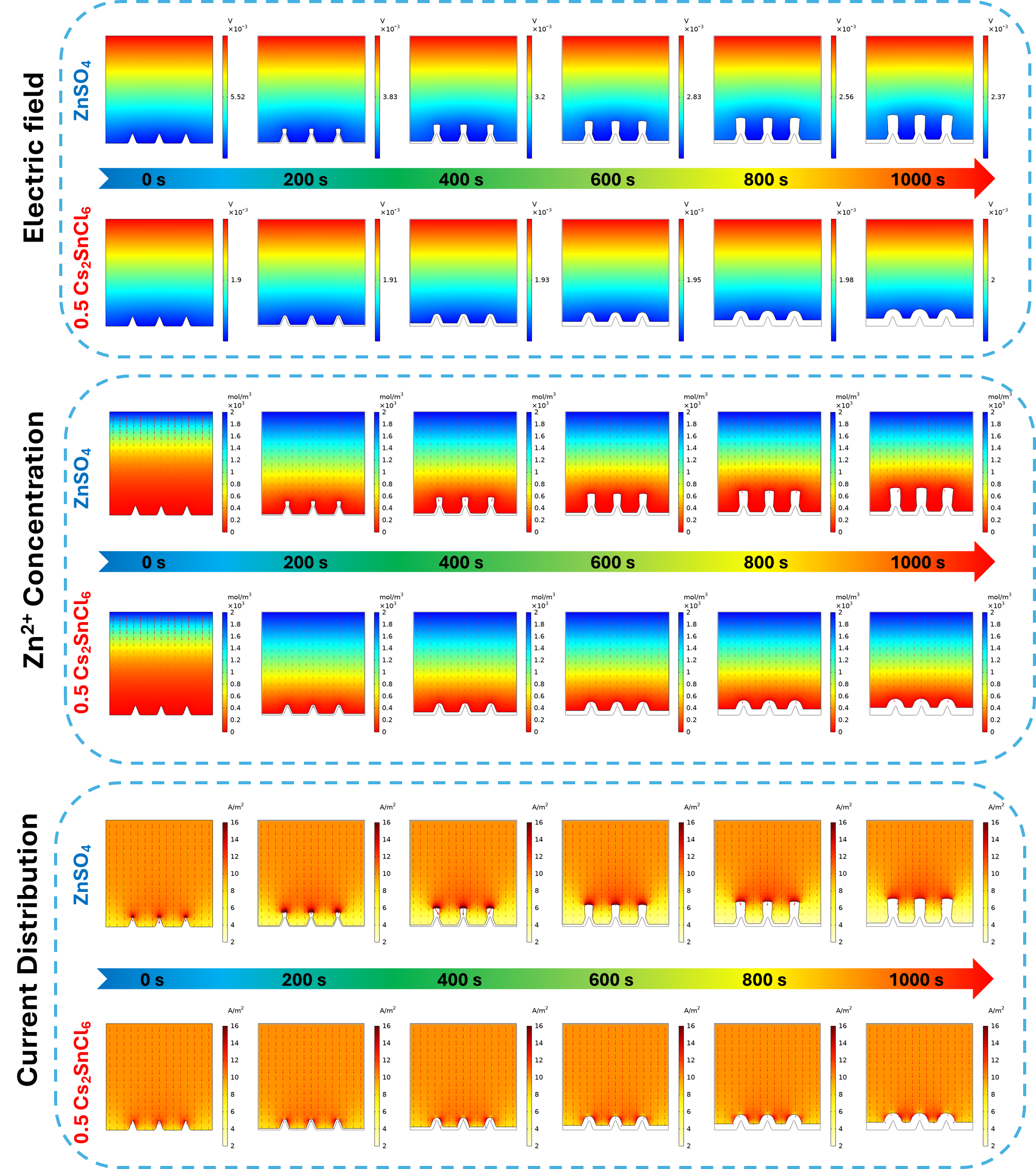


**Figure S19.** COMSOL simulation result of electric field, Zn^2+^ ion concentration, and current density distribution in 2 M ZnSO_4_ electrolyte and 0.5 Cs_2_SnCl_6_ electrolyte.


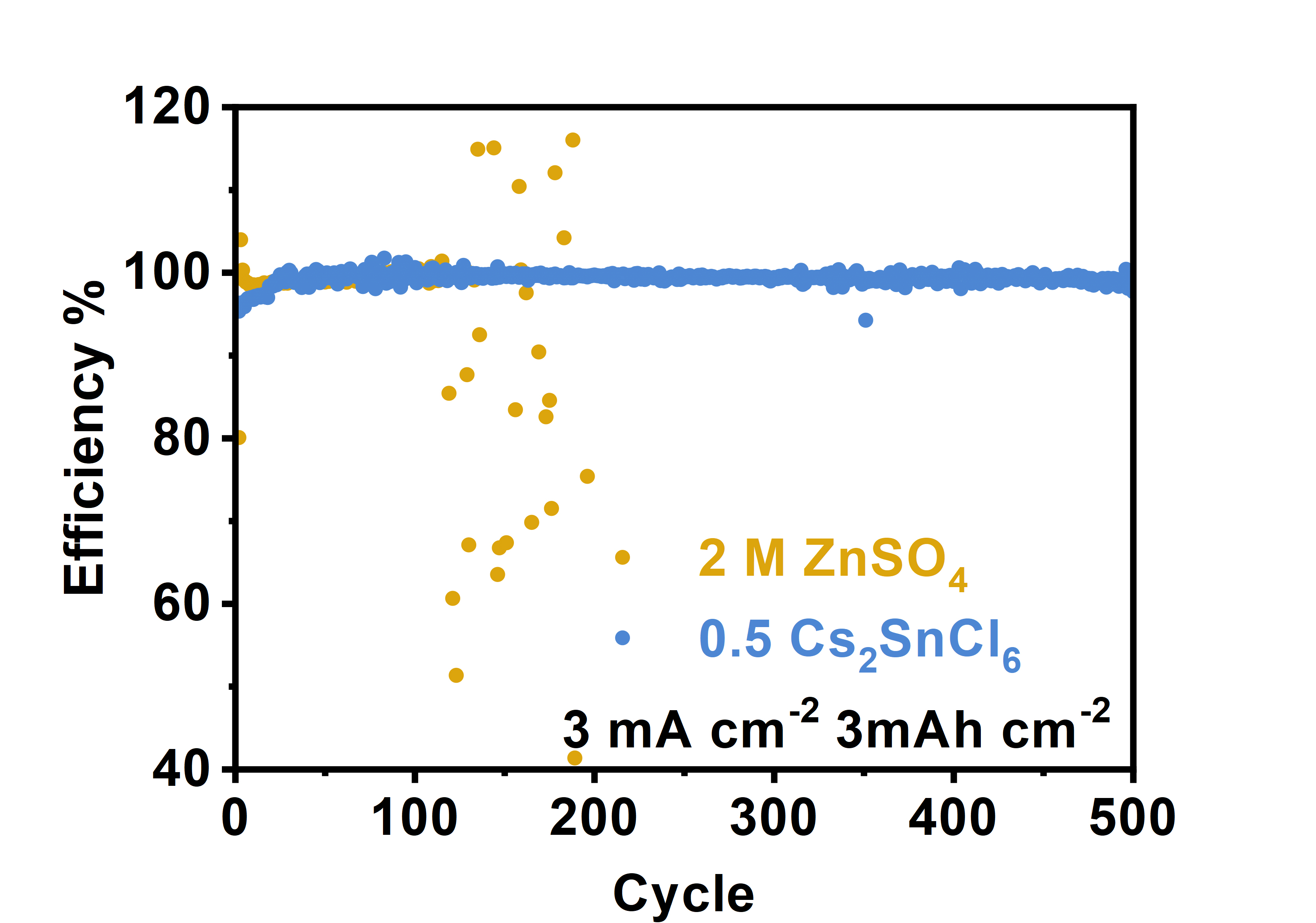


**Figure S20.** Cycling performance of the Zn||Cu cells at the current density of 3 mA cm^-2^ with the capacity limitation of 3 mAh cm^-2^ in the 2 M ZnSO_4_ electrolyte and 0.5 Cs_2_SnCl_6_ electrolyte.


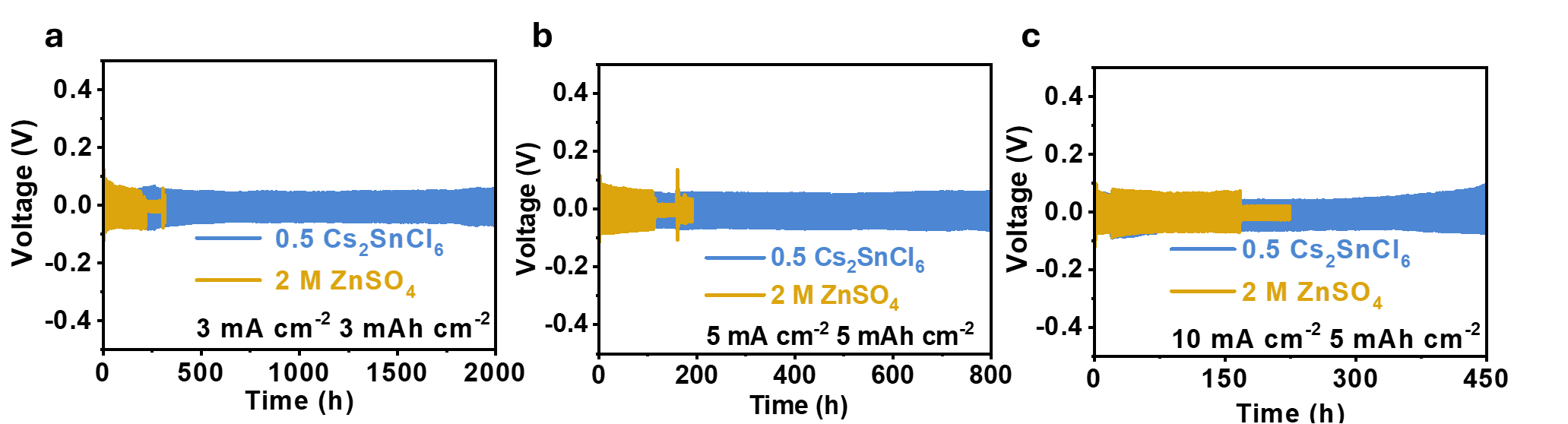


**Figure S21.** Cycling performance of the symmetric cells at different current densities and capacity limitations: (a) 3 mA cm^-2^, 3 mAh cm^-2^, (b) 5 mA cm^-2^, 5 mAh cm^-2^, and (c) 10 mA cm^-2^, 5 mAh cm^-2^.


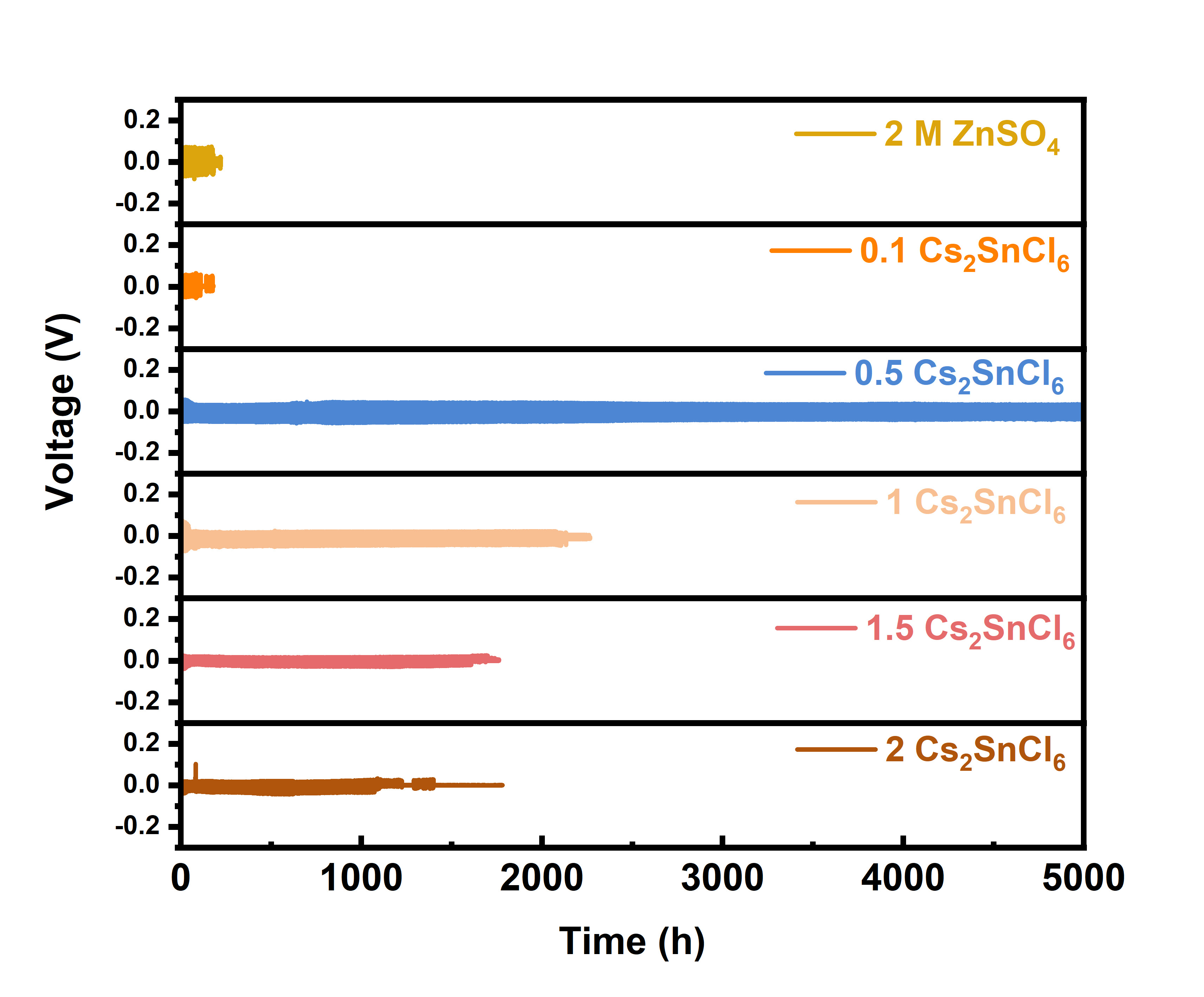


**Figure S22.** The cycling performance of Zn||Zn symmetric cells with different ratios of perovskite additives in the electrolytes**.**


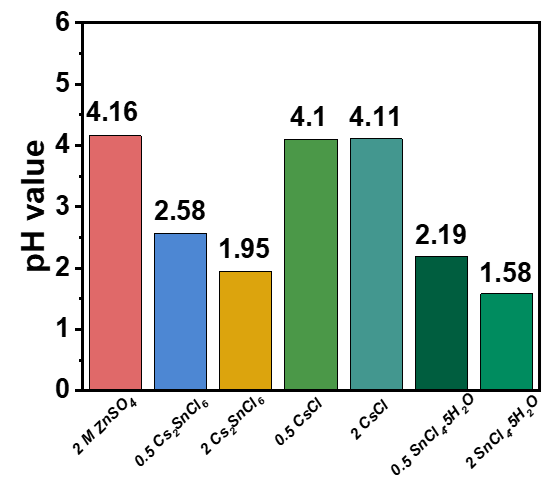


**Figure S23.** The pH values of ZnSO_4_ electrolytes with different concentrations of the Cs_2_SnCl_6_ additive.


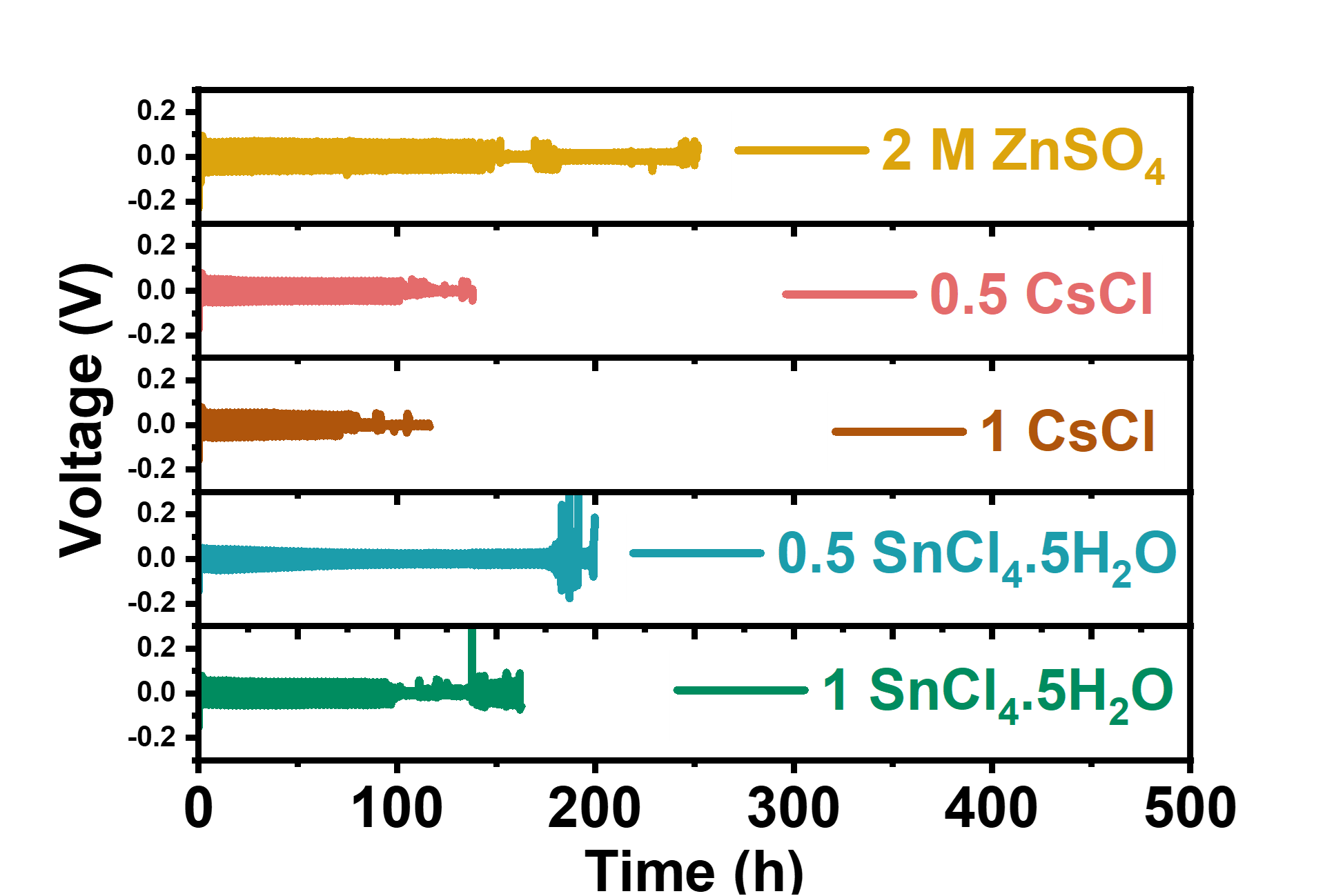


**Figure S24.** The cycling performance of the symmetric cells with different electrolyte additives under the current density of 1 mA cm^-2^ with the capacity limitation of 1 mAh cm^-2^.


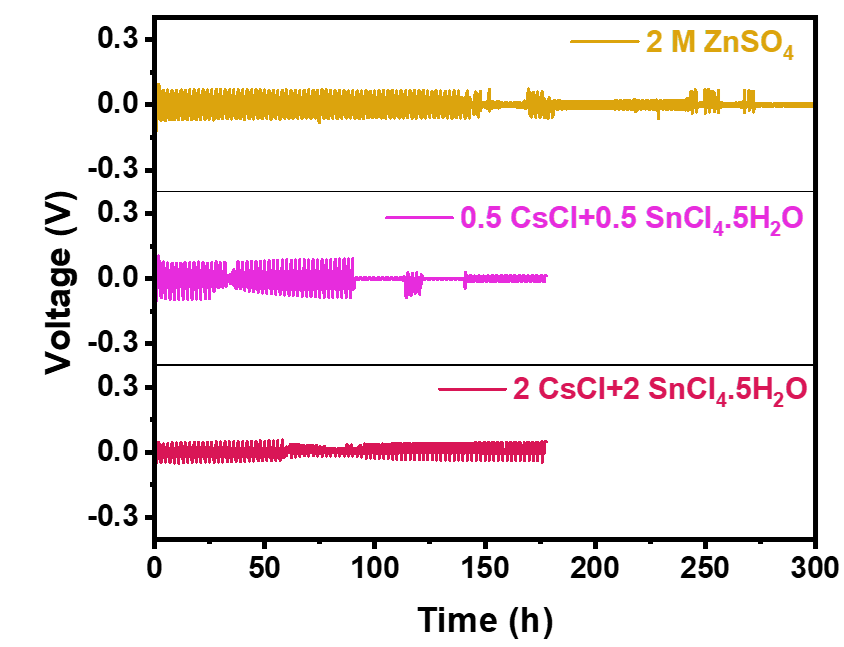


**Figure S25** The cycling performance of the symmetric cells with CsCl and SnCl_4_.H_2_O additives under the current density of 1 mA cm^-2^ with the capacity limitation of 1 mAh cm^-2^.

**
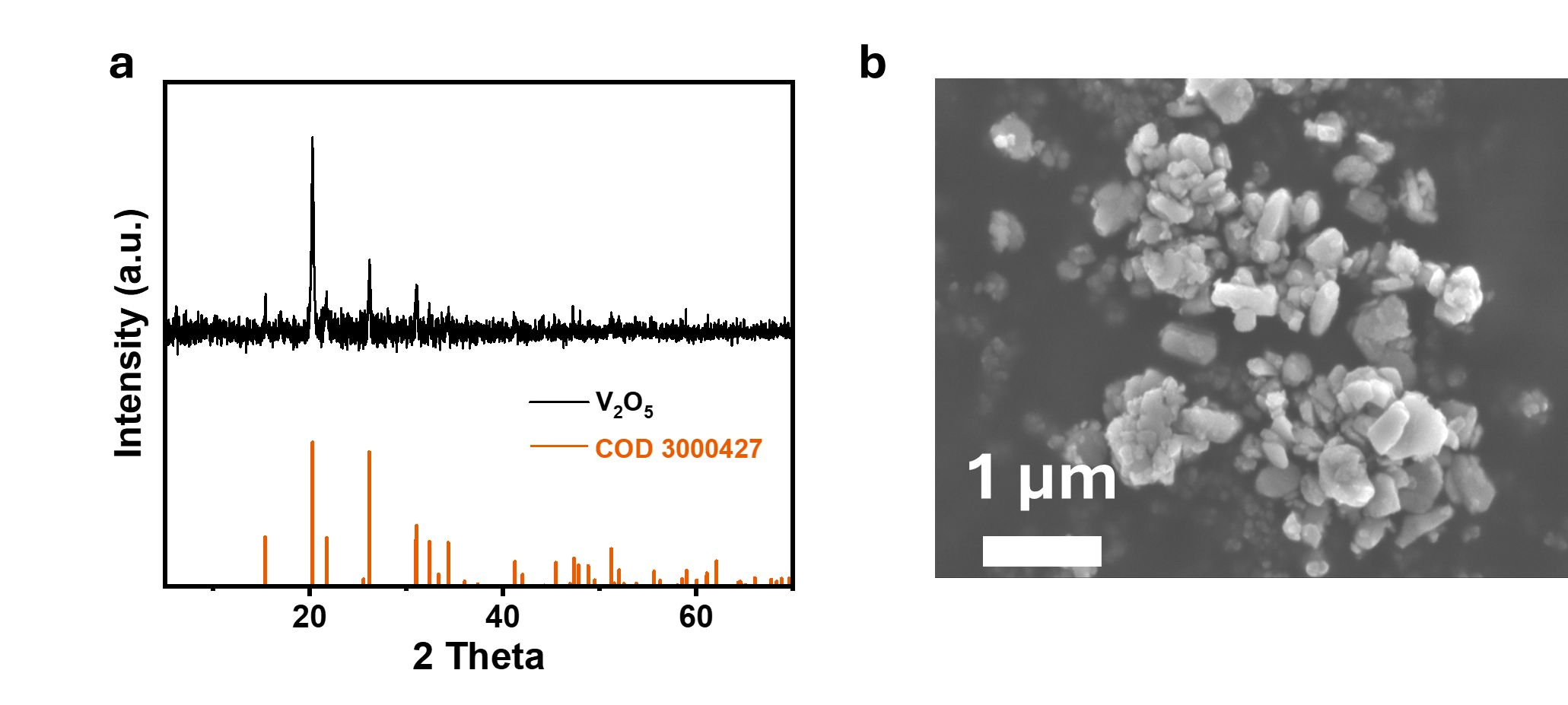
Figure S26** (a) XRD pattern of the commercial V_2_O_5_ powder, confirming phase purity and matching well with the reference data (COD 3000427). (b) SEM image of the commercial V_2_O_5_ powder with sizes in the micron range.

**
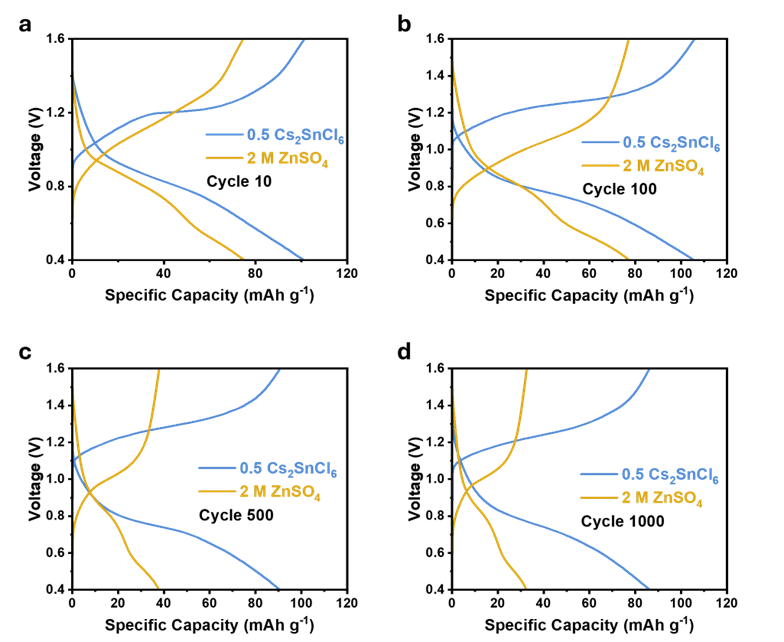
**

**Figure S27** GCD curves of Zn||V_2_O_5_ full cells using 0.5 M Cs_2_SnCl_6_ and 2 M ZnSO_4_ electrolytes at selected cycles: (a) 10th, (b) 100th, (c) 500th, and (d) 1000th. The Cs_2_SnCl_6_-containing cell exhibits higher capacity and better voltage profiles throughout cycling.
